# The CRTC-CREB axis functions as a transcriptional sensor to protect against proteotoxic stress in Drosophila

**DOI:** 10.1038/s41419-022-05122-y

**Published:** 2022-08-06

**Authors:** Youjie Yin, Peng Ma, Saifei Wang, Yao Zhang, Ruolei Han, Chunyu Huo, Meixian Wu, Hansong Deng

**Affiliations:** Yangzhi Rehabilitation Hospital, Sunshine Rehabilitation Center, School of Life Sciences and Technology, Tongji University, Shanghai, 20092 China

**Keywords:** Protein aggregation, High-throughput screening, Neurodegeneration

## Abstract

cAMP Responsible Element Binding Protein (CREB) is an evolutionarily conserved transcriptional factor that regulates cell growth, synaptic plasticity and so on. In this study, we unexpectedly found proteasome inhibitors, such as MLN2238, robustly increase CREB activity in adult flies through a large-scale compound screening. Mechanistically, reactive oxidative species (ROS) generated by proteasome inhibition are required and sufficient to promote CREB activity through c-Jun N-terminal kinase (JNK). In 293 T cells, JNK activation by MLN2238 is also required for increase of CREB phosphorylation at Ser^133^. Meanwhile, transcriptome analysis in fly intestine identified a group of genes involved in redox and proteostatic regulation are augmented by overexpressing CRTC (CREB-regulated transcriptional coactivator). Intriguingly, CRTC overexpression in muscles robustly restores protein folding and proteasomal activity in a fly Huntington’s disease (HD) model, and ameliorates HD related pathogenesis, such as protein aggregates, motility, and lifespan. Moreover, CREB activity increases during aging, and further enhances its activity can suppress protein aggregates in aged muscles. Together, our results identified CRTC/CREB downstream ROS/JNK signaling as a conserved sensor to tackle oxidative and proteotoxic stresses. Boosting CRTC/CREB activity is a potential therapeutic strategy to treat aging related protein aggregation diseases.

## Introduction

cAMP Responsible Element Binding Protein (CREB) belongs to the family of leucine zipper transcription factors that regulate various metabolic and developmental signals [1–3]. It receives signals from upstream signaling input, such as protein kinase A (PKA) and Calcium, and its activity is regulated by phosphorylation [4, 5]. Phosphorylated CREB translocates to the nucleus and is co-activated by CREB responsible transcriptional coactivator (CRTC) and CBP [6–8]. Activated CREB is then recruited to target genes containing CREB responsible elements (CRE) [4, 9]. In mammals, phosphorylation of CREB in Ser133 is critical for CREB-mediated transcription by facilitating its association with CREB-binding protein (CBP) and p300 [4, 8]. In addition to cAMP/PKA and Ca2+/calmodulin-dependent protein kinases (CaMKs), CREB can also be phosphorylated in response to multiple extra-cellular signals, such as growth factor induced MAPKs [10], insulin/Akt [11], and UV radiations [12]. However, the counterpart of Ser133 in fly CREB (dCREB2), Ser231, is predominantly phosphorylated under basal conditions, [13] and CREB activation is presumably determined by its nuclear abundance [14].

Recent studies have also indicated that CRTC, also known as TORC, is another evolutionarily conserved transcriptional co-activator of CREB [3, 6]. In response to cAMP and Calcium, CRTC was dephosphorylated by calcineurin and then translocate to nucleus and bind with CREB [6]. Moreover, we previously found as well that CREB with its co-activator, CRTC, regulates intestinal stem cell proliferation in response to elevated cytosolic Ca^2+^ in *Drosophila* [15]. A recent study also showed that CRTC(TORC) mutants were sensitive to oxidative stress, suggesting that CRTC was also involved in stress response [3].

Previously, a series of CREB agonists have been identified by high-throughput screenings in cell-based assays [16]. However, whether these compounds can increase CREB’s activity in vivo remains unknown. Poor solubility of compounds remains as a main obstacle for large-scale screening in adult flies [17, 18]. Nevertheless, we recently developed a sustainable delivery system, U-GLAD (U shape Gum Arabic Liquid Assisted Drug delivery system), which successfully tackled these issues [19]. Take advantage of this system, we unexpectedly found all proteasome inhibitors in FDA approved drug libraries can increase CREB’s activity in adult flies. The ubiquitin-proteasome system (UPS) is critical for protein turnover and degradation [20]. Proteasome inhibition leads to the accumulation of misfolded proteins, which can trigger the unfolded protein response (UPR) in ERs or mitochondria to alleviate the burden of misfolded protein by increasing the cells’ capacity for protein folding, degradation and transport processes [21].

In addition to ER stress, proteasome inhibition also generates ROS largely due to mitochondrial dysfunction [22–24]. Although generally considered as detrimental, emerging evidence showed that ROS is an important signaling molecule for inflammation and proliferation [25, 26]. Recently, we also showed that excessive ROS suppressed differentiation of intestinal progenitor/stem cells through the JNK cascade [27]. JNK and p38 are both stress related MAPK kinases, which can be activated by ROS and ER stresses [28, 29].

Protein misfolding and aggregation are hallmarks of multiple neurodegenerative diseases, although the disease-related dynamic nature of aggregates is not fully determined [30–33]. For instance, protein aggregates due to expansions of poly-Q repeats within exon 1of the huntingtin (HTT) gene cause the Huntington’s disease (HD), which is an incurable neurodegenerative disease characterized by abnormal motility and early death [34]. Studies also showed that huntingtin aggregates can interfere with transcription by sequestering CBP, a histone acetylase, in the cytosol [35], while histone-deacetylase inhibitor (HDAC inhibitors) can reduce pathogenesis in a fly HD models [36]. However, as a CREB coactivator, what aspect(s) of CBP/CREB transcription can regulate huntingtin aggregates, however, remains elusive.

Here, we demonstrated that proteosome perturbation activated CREB through the ROS/JNK cascade, and this cascade is also conserved in mammals. CRTC/CREB axis can upregulate the transcription of molecular chaperones, as well as proteasome subunits. Furthermore, CRTC overexpression also reduced protein aggregates and lethality in a fly HD model. Together, our results showed CRTC/CREB as a novel redox sensor that regulates stress responses and proteostasis, thereby highlighting its role in aging and neurodegenerative diseases.

## Results

### A large-scale screening identified proteasome inhibitors promote CREB activity in adult flies

**Fig. 1:**
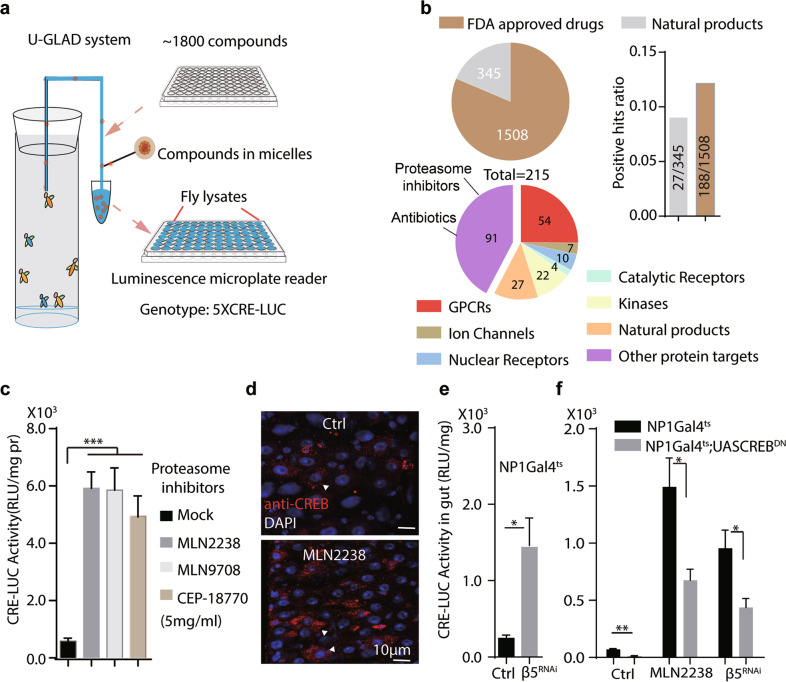
A large-scale screening identified proteasome inhibitors as CREB activators. **a** Schematics of a compound screening in adult flies. Compounds were mixed in gum Arabic (GA) micelles and delivered to flies by U-GLAD (U-shaped GA liquid-assisted delivery) system. In brief, compound powder was grinded with gum Arabic and dissolved in chemical defined liquid food at 5 mg/ml. Drug containing liquid food was delivered to the flies through a U-shaped glass capillary by siphoning. Details refer to the main text and the method section. **b** Summary of compound screening results. A compound library contains 1853 FDA approved drug library and 215 natural products was screened. Classification of the positive hits (those with more than 5-fold increase of CRE-LUC) in the library were presented. **c** Proteasome inhibitors promote CREB activity in adult flies. Compounds were fed at 5 mg/ml for 24 h. One-way ANOVA analysis for statistics, ***P < 0.001. CRE-LUC activity was normalized with total protein content. **d** CREB expression was examined in fly intestine by anti-CREB staining. DAPI counterstains nuclei in blue. Arrowheads denotes typical CREB signals in ECs. Scale bar: 10 μm. **e** Intestinal CRE-LUC activity was examined when proteasome subunit *prosβ5* was inhibited in enterocytes at 29 °C for 4 days. Student’s *t*-test performed for statistics. *P < 0.05. *n* = 15 for each condition. Genotype: *NP1-Gal4*^*ts*^*, UAS-prosβ5*^*RNAi*^. **f** Intestinal CRE-LUC activity induced by MLN2238 or β5RNAi is suppressed by CREB^DN^. Student’s *t*-test performed for statistics. *P < 0.05, **P < 0.01, *n* = 15 for each condition. Genotypes: *NP1-Gal4*^*ts*^*; UAS-prosβ5*^*RNAi*^ and *NP1-Gal4*^*ts*^*; UAS-CREB*^*DN*^*, UAS-prosβ5*^*RNAi*^.

To identify CREB modulators in adult flies, we conducted a large-scale screening using the CRE-LUC reporter as a readout of a compound library containing 1508 FDA approved drugs and 345 natural products (DiscoveryProbe^™^ FDA-approved Drug Library, ApexBio Cat# L1021, Supplemental table S1). The compounds were mixed in gum Arabic and dissolved in chemically defined liquid food [37] to form micelles. The product was sustainably delivered to adult flies via the U-GLAD system [19] **(**Fig. 1a**)**. After a 24 h feeding, around 11.6% (215/1853) of the drugs (final concentration 5 mg/ml) showed at least a 5-fold increase of CRE luciferase activity in whole fly lysates (Fig. 1b). Among them, the FDA approved drugs showed a slightly higher positive rate than natural compounds (~12% in FDA proved drugs *v.s*. ~8% for natural products) (Fig. 1b, and Supplementary Table 1). Inhibitors of PDE (Roflumilast) and GPCRs (54/215), which are typical regulators of CREB [38], are among the positive hits, verifying the success of the screening(Fig. 1b, and Supplementary table 1). Intriguingly, proteasome inhibitors, such as MLN2238, MLN9708, and CEP-18770, potently increased CRE-Luc activities in flies extracts (5 mg/ml, 24 h) (Fig. 1c). CRE-Luc activities are higher in the brain and abdominal adipose tissues than in the intestines and thoraces, which can all be further increased by MLN2238 (5 mg/ml, 24 h) also increased CRE-LUC levels in all tissues (Supplementary Fig. 1a). Notably, the effect of MLN2238 on CRE-LUC correlated with its concentrations, whereas further increase from 5 mg/ml to 10 mg/ml did not show significant increase in CRE-Luc activities (Supplementary Fig. 1b). Additionally, MLN2238 at 5 mg/ml did not affect viability or food intake rates as indicated by CAFÉ assay and by dyed food consumption (Supplementary Fig. 1c, d and data not shown). In contrast, antibiotics as another group of positive hits (such as Roxithromycin (ROX) and Ornidazole (ONZ)) substantially reduced food intake, mimicking the effect of starvation on CRE-LUC activities (Supplementary Fig. 1c, f). Subsequently, the effect of MLN2238 on CREB activity was further verified using a commercial antibody against CREB [39]. In adult fly guts, CREB was highly expressed in enteroendocrine cells (revealed by anti-Prospero staining, Supplementary Fig. 1g). Their expression in progenitor cells (GFP + cells where UASGFP driven by esgGal4) or in enterocytes (the main differentiated cell type) is barely detectable (Supplementary Fig. 1g). However, strong CREB signals in enterocytes were observed after MLN2238 treatment (Fig. 1d), which was abolished in FLP-out clones of CREB^RNAi^, indicating these signals indeed reflect CREB (Supplementary Fig. 1h).

Previous results have shown that the target of MLN2238 was Prosβ5 subunit, the core catalytic subunit associated with the chymotrypsin-like proteolytic activities [40]. Genetically silencing the *Prosβ5* subunit (CG12323, herein refereed as *β5*) in enterocytes using the binary UAS-GAL4 system via *NP1-GAL4; UAS-β5*^*RNAi*^ significantly increased intestinal CRE-Luc activity (Fig. 1e). In contrast, overexpressing a repressor form of CREB, *dcreb-2* [2] (refereed as CREB^DN^ herein), eliminated CRE-LUC levels increased by MLN2238 or by β5 knock-down in enterocytes (Fig. 1f). Together, these results indicated that proteosome inhibition boosted CREB activity in adult flies.

### Proteasome inhibition enhances CREB activity through ROS

**Fig. 2:**
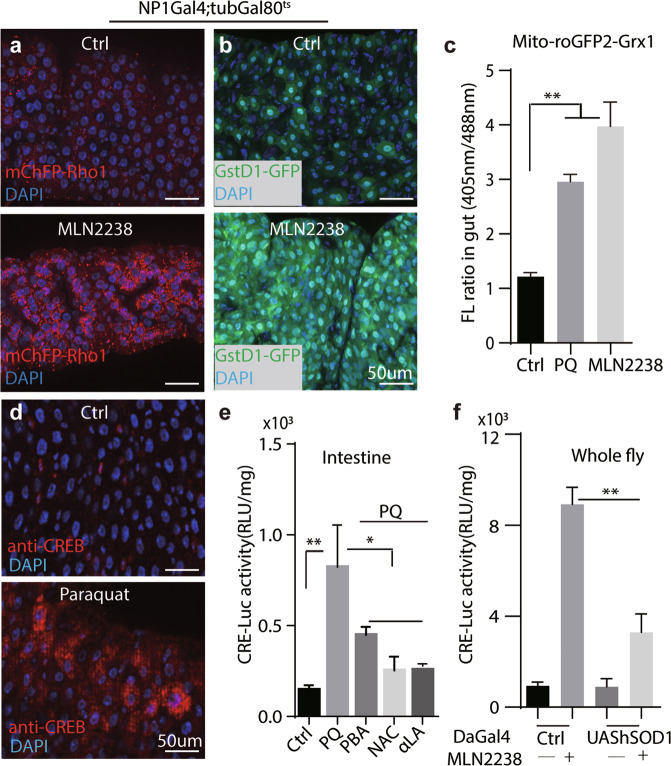
Proteasome inhibition regulates CREB activity through Reactive Oxidative Species (ROS). **a** Proteostatic activity in fly intestine was examined with mCherry-RFP-Rho1(in red) after MLN2238 treatment (5 mg/ml, 24 h). DAPI counterstains nuclei in blue. Genotype: *NP1-Gal4, tubGal80*^*ts*^*; mChFP-Rho1*. **b** Oxidative stresses was monitored by a genetic reporter, *GstD1-GFP*. Genotype: *NP1-Gal4*^*ts*^*; GstD1-GFP*. **c** Mitochondrial ROS in intestine was measured and quantified by a fluorescent ratio-metric sensor *Casper-mito-roGFP2-Grx1*. Probe fluorescence was excited sequentially at 405 and 488 nm and detected at 500–530 nm. *n* = 6 for each condition. Genotype: *Casper-mito-roGFP2-Grx1*. Student’s *t*-test performed for statistical analysis. **P < 0.01. **d** CREB protein level after paraquat (PQ) treatment was detected by immunostaining against CREB (red), DAPI counterstains nuclei (blue). Genotype: *NP1-Gal4, tubGal80*^*ts*^. Representative images (8 animals of each condition) are shown. **e** The effect of antioxidants on CRE-LUC activity was quantified after paraquat (PQ) treatment. PBA: 4-Phenylbutyric acid, NAC: N-acetylcysteine, αLA: α lipoic acid. Antioxidants were fed at 5 mg/ml for 5-8 days before analysis. One-way ANOVA analysis performed for statistics. *P < 0.05, **P < 0.01. **f** CRE-LUC activity was analyzed in whole fly extracts with indicated genotypes. **P < 0.01, Student’s t-test for statistical analysis. *n* = 6 for each condition. Scale bars: 50 μm for panels **a–d**.

Treatment with MLN2238 or by β5 knock-down in enterocytes profoundly blocked intestinal proteostasis, as indicated by mCherry-RFP-Rho1, a genetic reporter for proteasome capacity [41, 42] and accumulation of ubiquitinated proteins (detected by FK2 antibody) (Fig. 2a and Supplementary Fig. 2a, c).

Proteasome impairment cause the buildup of misfolded proteins in the ER, leading to ER stress [43]. Indeed, MLN2238 treatment caused ER stresses as indicated by the accumulation of phospho-eIF2α staining in guts [44] (Supplementary Fig. 2b, c). However, blockage of ER^UPR^ by knocking down *PERK* in enterocytes did not increase CRE-LUC level, while increasing phospho-eIF2α staining in progenitors cell-non autonomously [44] (Supplementary Fig. 2b–d). Impaired activity of sarco-endoplasmic reticulum Ca^2+^ ATPase (SERCA) causes ER stresses while also increasing cytosolic Ca^2+^ [45]. Elevated cytoCa^2+^ can dephosphorylate CRTC and promote its nuclear entry through calcineurin [15]. Indeed, knocking down *SERCA* in enterocytes by NP1-Gal4; UAS::*SERCA*^RNAi^ is sufficient to increase CRE-LUC activity, as well as ER stresses (indicated by anti-peIF2α staining, Supplementary Fig. 2f, g). However, *SERCA*^RNAi^ induced CRE-LUC is blocked by Crtc^RNAi^, while ER stresses remains high (Supplementary Fig. 2f, g), suggesting that *SERCA*^RNAi^ induced CRE-LUC is mainly due to elevated cytoCa^2+^ instead of ER stress.

ER oxidoreductin 1–like (Ero1L) is an ER located oxidoreductase that promotes protein folding by catalyzing the protein disulfide bond formation while facilitating ROS production [44, 46]. Similarly, Ero1L knock-down in enterocytes by *NP1-Gal4*^*ts*^*/UAS- Ero1L*^*RNAi*^ failed to increase CRE-LUC levels either (Supplementary Fig. 2d). Together, these results indicated that MLN2238-induced CREB activity is less likely caused by ER stress.

Proteasome inhibition is often compensated by autophagic degradation [47, 48]. Studies also showed that Ca^2+^ homeostasis was also altered by the proteasome inhibition [49]. However, MLN2238 treatment didn’t show significant changes in autophagic activities (revealed by UAS-LC3-GFP) [50] or cytosolic Ca^2+^ levels (revealed by UAS-GCaMP5) [51, 52] (Supplementary Fig. 3a, b).

Additionally, proteasome dysfunction in flies also increased ROS production due to mitochondrial proteome damage [22]. Indeed, we found intestinal ROS levels were upregulated by MLN2238 as indicated by GstD1:GFP (Fig. 2b), a genetic indicator of ROS [53]. Mitochondrial ROS indicated by *Casper-mito-roGFP2-Grx1* [34] was also significantly increased by MLN2238 (Fig. 2c and Supplementary Fig. 3c). Treatment with paraquat (PQ), a widely used herbicide and potent ROS inducer, also increased ROS and intestinal CREB activity (Fig. 2c–e and Supplementary Fig. 3d). In contrast, feeding with antioxidants, such as N-acetylcysteine (NAC) (1 mg/ml, 8days), strongly reversed PQ-induced CRE-LUC in a dosage dependent manner (Fig. 2e and Supplementary Fig. 3e). Similar rescue results were also obtained with other antioxidants, such as 4-Phenylbutyric acid (PBA), and α-lipoic acid (αLA) (2 mM, 8days) (Fig. 2e). Furthermore, genetically overexpressing human superoxide dismutase 1 (hSOD1) [54] also significantly suppressed MLN2238 induced CRE-LUC levels (Fig. 2f). Together, these results indicated that elevated ROS levels were necessary and sufficient to activate CREB after proteasome inhibition.

### JNK is downstream of proteasome inhibition to regulate CREB activity

**Fig. 3:**
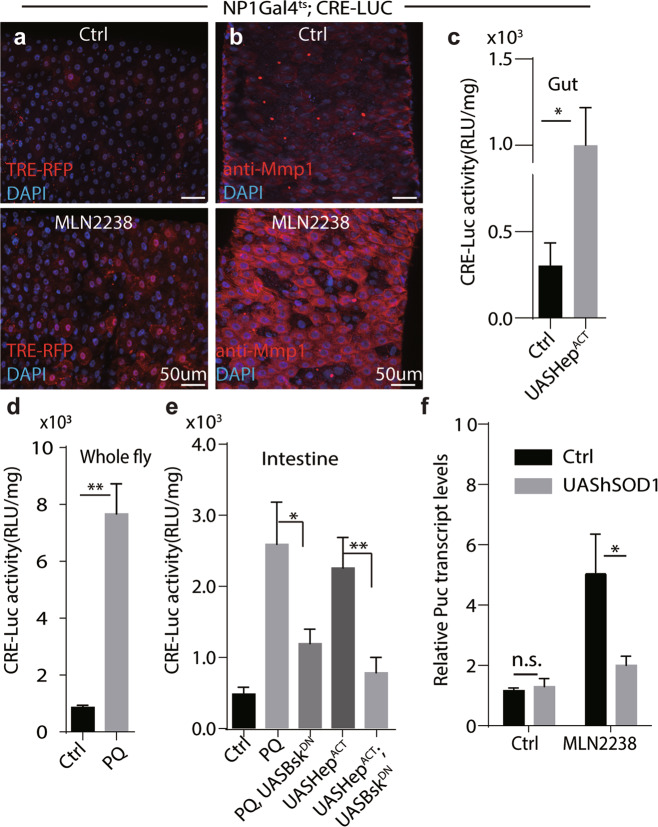
JNK functions downstream of proteasome inhibition to regulate CREB activity. JNK activity in fly guts was upregulated after MLN2238 treatment as revealed by TRE-RFP (**a**) and by anti-Mmp1 staining (**b**). Scale bars for **a**, **b**, 50 μm. **c–e** CRE-LUC activity was analyzed under indicated conditions. Genotypes for **c** *NP1-Gal4*^*ts*^*, 5xCRE-LUC*(ctrl) or *NP1-Gal4*^*ts*^*, 5xCRE-LUC, UAS-Hep*^*ACT*^, **d** *NP1-Gal4*^*ts*^*, 5xCRE-LUC;* **e** *NP1-Gal4*^*ts*^*, 5xCRE-LUC* (ctrl), *NP1-Gal*^*ts*^*, 5xCRE-LUC, UAS-Bsk*^*DN*^ *and NP1-Gal4*^*ts*^*, 5xCRE-LUC; UAS-Hep*^*ACT*^*, UAS-Bsk*^*DN*^. Student’s *t*-Test for statistical analysis. **P < 0.01, *P < 0.05. **f** Relative transcripts of *puckered* was quantified under indicated conditions. *Rp49* was used as an internal control. Experiments were done in triplicates, *P* < 0.01, Student’s *t*-test for statistical analysis. Genotypes: *NP1-Gal4*^*ts*^*, 5xCRE-LUC* (ctrl) and *NP1-Gal4*^*ts*^*, UAS-hSOD1*.

ROS have been reported to activate stress related MAPKs, such as ERKs, JNKs, and p38 [55]. While p38 activities (detected by anti-phosphorylated p38 antibody) after MLN2238 treatment remained largely unchanged in fly guts (Supplementary Fig. 4a), JNK activities (revealed by *TRE*-RFP reporter [56], Mmp1 immunostaining [57], and transcription of *puckered*(puc) [58]) in enterocytes were significantly induced by MLN2238 or PQ (Fig. 3a, b and Supplementary Fig. 4b, c). Hemipterous (*Hep*) is an MAPKK that phosphorylates *Basket (Bsk)*, the fly JNK homolog [59]. As with PQ, overexpressing the constitutively active form of Hep (Hep^ACT^), increases CRE-Luc levels **(**Fig. 3d, e**)**. On the other hand, PQ or *Hep*^*ACT*^ induced CRE-Luc activation is significantly suppressed by *Bsk*^*DN*^, a dominant negative form of *Bsk* (Fig. 3e). Moreover, JNK activities (indicated by puckered transcript) induced by MLN2238 were robustly suppressed in flies with hSOD1 overexpressing in enterocytes. (Fig. 3f). Together, these results indicated that JNK signaling function downstream of ROS to regulate CREB activity.

### CRTC/CREB is essential to maintain redox and proteostatic homeostasis

**Fig. 4:**
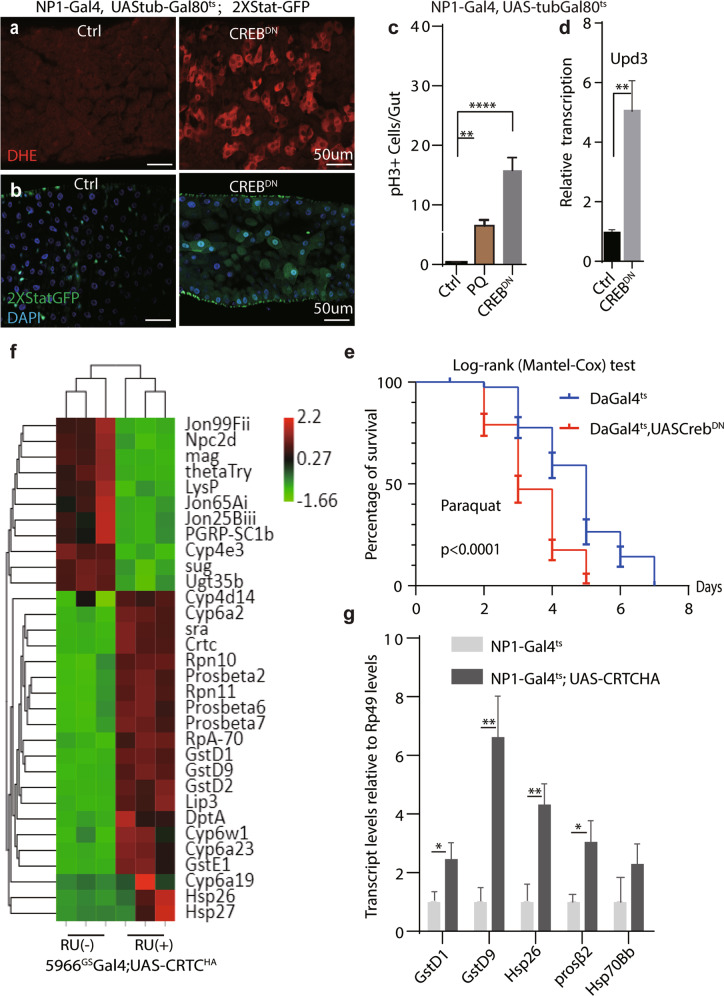
CRTC-CREB signaling is essential to maintain redox and proteostatic homeostasis. **a** ROS level in guts measured by DHE ex vivo live imaging. **b** JAK/STAT pathway activity monitored by *2xStat-GFP* reporter. Genotypes for **a**, **b** *NP1-Gal4, UAS-tubGal80*^*ts*^*;2xStat-GFP*. Scale bars: 50 um. *n* = 6 for each condition. **c** Mitotic ISCs were quantified by anti-phospho-histone H3 staining. *n* = 12 for each condition. One-way ANOVA for statistical analysis. ****P < 0.0001. **d** Relative upd3 transcripts was quantified in guts by RT-qPCR. Student’s *t*-test for statistical analysis. **P < 0.01. Samples were run in triplicates. **e** Survival curve of female flies after paraquat treatment. Log-rank (Mantel-Cox test) for analysis. *n* = 60 flies for each condition. *P* < 0.0001. **f** Clustered heatmap analysis of genes changed by CRTC overexpression. Genes involves in oxidative stress response, chaperones and proteasome subunits are significantly upregulated by CRTC overexpression. RU (-): Mock condition, RU (+): RU486 induction. Genotype: 5966-GS-GAL4, UAS-CRTC-HA. **g** Relative transcripts of genes was quantified by RT-qPCR and normalized with *Rp49*. Genotypes: *NP1-Gal4*^*ts*^*; UAS-CRTC-HA*. Samples were run in triplicates. Student’s t-test for statistical analysis. **P < 0.01. *P < 0.05.

Aforementioned results indicated that CREB can be activated by proteostatic and oxidative stresses. Therefore, we explored the role of CREB in proteostatic regulation. Expressing CREB^DN^ in fly intestine caused extensive ROS accumulation, as measured by Dihydroethidium (DHE) staining (Fig. 4a). ROS-mediated JNK activation also promoted the expression of the Unpaired (*Upd*) cytokines, which triggers ISC proliferation through paracrine JAK/STAT signaling [60]. Indeed, JAK/STAT activity (detected by *2XStatGFP* and transcription of *Upd3)* and number of mitotic ISCs (phospho-Histone 3 positive, pH3+) was significantly increased in *NP1-Gal4*^*ts*^*; UAS-CREB*^*DN*^ flies, as well as in flies fed with MLN2238 (5 mg/ml, 24 h) (Fig. 4b, d and Supplementary Figs. 4d and 5a).

Furthermore, flies with systematic CREB suppression (*Da-Gal4*^*ts*^*; UAS-CREB*^*DN*^) were sensitive to PQ and MLN2238 insults, and lived significantly shorter than the controls (Fig. 4e and Supplementary Fig. 5b). Similar results were obtained as well in heterozygous CRTC(*TORC*^*25-3*^*/+*) flies after PQ treatment (Supplementary Fig. 5c).

Alternatively, transcriptome analysis indicated that the genes involved in proteasome assembly, redox regulation, and protein folding were highly enriched among those differentially expressed genes (DEGs) in CRTC overexpressing (CRTC^OE^) intestines (Fig. 4f, Supplementary Fig. 5d, and Supplementary Table 2). Intriguingly, clustered heatmap analysis showed that stress response genes, such as *Hsp26*, *prosβ2* and *GstD1*, were also upregulated by CRTC^OE^, which was verified by RT-qPCR experiments (Fig. 4f, g). Moreover, many of these genes contains CRE sites near the transcriptional start site, and are highly enriched in ChIP-seq dataset for antibody against CREB in fly brain (Supplementary Fig. 5e) [61]. Together, these results indicated that CRTC/CREB is a novel modulator to prevent proteotoxic and oxidative stresses in fly gut.

### JNK is required for MLN 2238 mediated CREB activation in 293 T cells

**Fig. 5:**
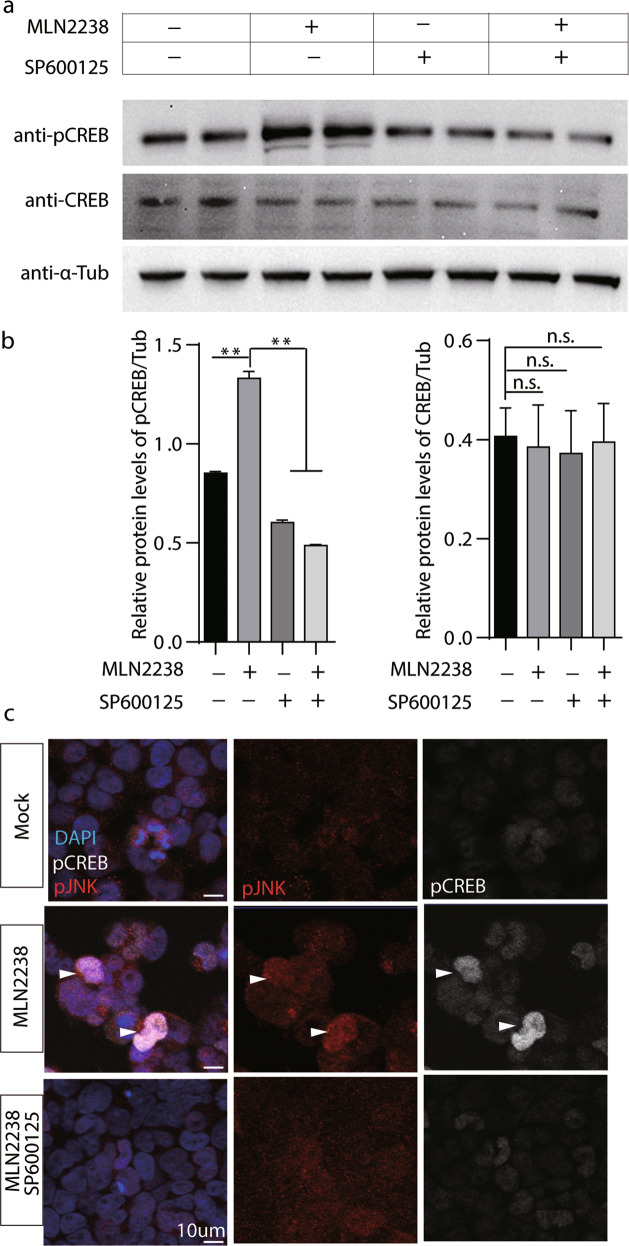
MLN 2238 activates JNK which is required for CREB activation in 293 T cells. **a** CREB phosphorylation status (Ser131) in 293 T cells was detected by Western blot. Total protein lysate from control and cells treated with MLN2238 (5 nM) and/or SP600125 (40 uM) for 24 h by immunoblotting using pCREB, CREB and α-tubulin antibodies. Representative blots from three independent experiments are shown. **b** Relative expression of CREB and pCREB in immuno-blots shown in panel a were quantified using α-tubulin as internal controls. *t*-Test was performed for statistical analysis. **p < 0.01, n.s.: no significance. **c** Representative images of immune-staining against phospho-JNK(pJNK at Thr^183^ and Tyr^185^) and phospho-CREB(anti-CREB Ser^133^) after treat with MLN2238 (5 nM) and/or SP600125 (40 uM) for 24 h in 293 T cells. Separate channels shown on the right. Scale bar: 10um. Arrowhead points to cells accumulated with pCREB and pJNK after MLN2238 treatment.

Next, we sought to test whether proteasome inhibition can also activate CREB in mammalian system. Intriguingly, phospho-CREB(Ser^133^) is robustly increased after MLN2238 treatment at 5 nM for 24 h, although total CREB protein level is largely unchanged (Fig. 5a, b**)**. Immunostaining results indicated that JNK is dispersed in cytosol under mock condition, which form large cytosolic foci after MLN2238 treatment (Supplemental Fig. 6). Intriguingly, simultaneously treatment with a JNK specific inhibitor, SP600125 [62] at 40uM for 24 h significantly reduces phospho-CREB level (Fig. 5a, b**)**. Moreover, phospho-CREB (Ser133) is drastically increased in nucleus after MLN2238 treatment, which was significantly reduced by SP600125 (Fig. 5c). These results indicated that JNK is required for MLN2238 mediated CREB activation in 293 T cells.

### Increasing CREB activity rescues pathogenesis in a fly model of Huntington’s disease

Proteostatic dysfunction is a common feature for many neurodegenerative diseases [63]. Expanded polyglutamine repeats in the Huntington (Htt) protein have been shown to be prone to form toxic aggregates. Likewise, flies overexpressing Htt exon 1 fragment with Q120 repeats (*UAS-HTT.ex1.Q120*) recapitulated multiple pathological defects in HD patients, including protein aggregates and early death [64, 65]. Proteasome failure in fly indirect flight muscles (IFMs) is an early sign of tissue aging [66]. Therefore, we sought to establish a HD model in IFMs and test whether increasing CREB activity can suppress HD related pathogenesis.

**Fig. 6:**
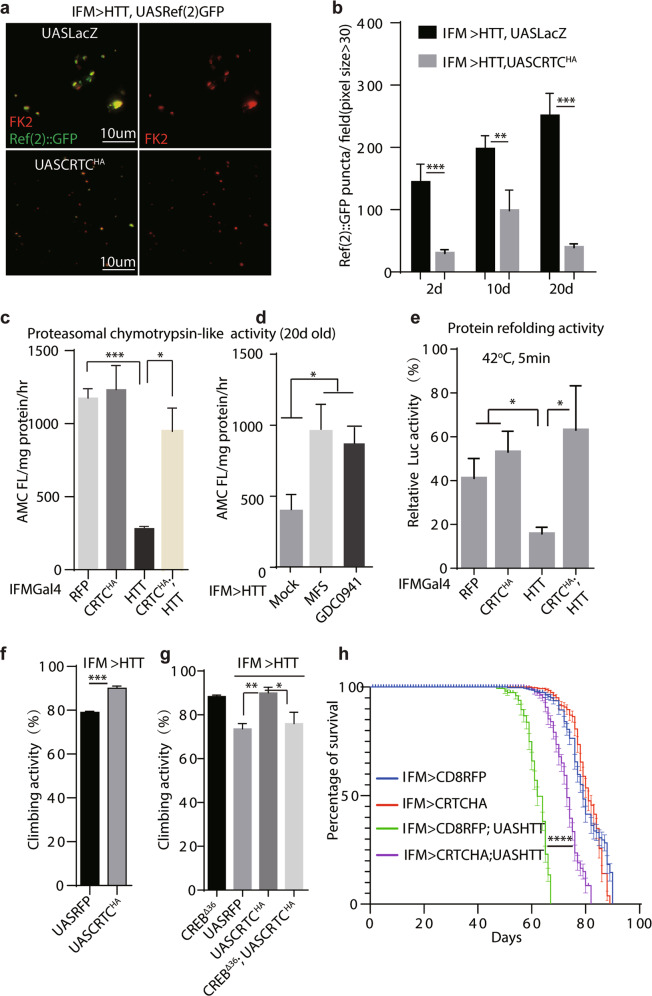
Increasing CREB activity ameliorates pathogenesis in a fly model of Huntington’s disease. **a** Ubiquitinated proteins in indirect flight muscles detected by anti-FK2 staining. Representative images were shown, Ref2P::GFP (green) and FK2(grey). Separate FK2 channel was shown on the right. *n* = 12 for each condition. Genotypes: *IFM-Gal4, UAS-HTT.ex1.Q120; UAS-Ref(2)P::GFP, UAS-LacZ or IFM-Gal4, UAS-HTT.ex1.Q120; UAS-Ref(2)P::GFP, UAS-CRTC:HA*. Scale bars: 10 um. **b** *Ref(2)P::GFP* positive puncta with pixel size bigger than 30 was quantified at different timepoints. *n* = 9 for each condition. Student’s *t*-test for statistical analysis. **p < 0.01. ***p < 0.001. Genotypes for **a**, **b** *IFM-Gal4, UAS-HTT.ex1.Q120; UAS-Ref*(*2*)*P::GFP, UAS-LacZ or IFM-Gal4, UAS-HTT.ex1.Q120; UAS-Ref(2)P::GFP, UAS-CRTC:HA*. **c** Chymotrypsin-like proteasomal activity (measured with fluorescence Suc-LLVY-AMC) in thoraces was quantified. AMC fluorescence in an hour was normalized with protein level. Student’s t-test for statistical analysis. *P < 0.05. ***P < 0.001. 8 thoraces were tested for each condition. **d** Chymotrypsin-like proteasomal activity in thoraces of 20d-old flies was quantified after treatment with miltefosine (MFS) (5days, 5 mg/ml) or GDC0941 (3days, 2 mg/ml). Student’s *t*-test for statistical analysis. **P < 0.01. 10 thoraces were tested for each condition. **e** Protein refolding capacity monitored by luciferase recovery after denatured at 42 °C for 5 mins. Experiments were performed in triplicates. Student’s *t*-test for statistical analysis. *P < 0.05. 10 thoraces were tested for each condition. **f**, **g** Climbing ability of 20d-old flies was examined with indicated genotypes. Student’s *t*-test for statistical analysis. Females were tested in **f** and male tested in panel **g**. ***p < 0.001. *n* = 25 for each condition. **h** Lifespan of female flies with indicated genotypes. *n* = 50-65 for each condition. Three independent replicates were measured. Log rank was performed for statistical analysis. ****p < 0.0001 between flies with genotypes *IFM-Gal4, UAS-HTT.ex1.Q120; UAS-CD8::RFP* and *IFM-Gal4, UAS-HTT.ex1.Q120; UAS-CRTC:HA*. Genotypes for **c–g** *IFM-Gal4, UAS-HTT.ex1.Q120; UAS-CD8::RFP* or *IFM-Gal4, UAS-HTT.ex1.Q120; UAS-CRTC:HA*, or *IFM-Gal4; UAS-CD8::RFP* or *IFM-Gal4; UAS-CRTC:HA*.

Indeed, overexpressing *Httex1Q120* in IFMs by IFMGal4 [67] progressively induced protein aggregates as indicated by Ref(2)P::GFP positive puncta (Fig. 6a, b and Supplementary Fig. 7a). Ref(2)P is the *Drosophila* orthologue of the mammalian p62 that colocalizes with ubiquitinated proteins [68]. Intriguingly, these Ref(2)P::GFP positive punctate in *Httex1Q120* IFMs were significantly reduced when CRTC was simultaneously overexpressed (Fig. 6b). Consistently, ubiquitin positive protein aggregates in Triton-insoluble fractions from *IFM-Gal4/UAS-Httex1Q120* thoraces extracts are significantly reduced by CRTC overexpression (Supplementary Fig. 7b).

The proteasomal chymotrypsin-like activity examined with fluorescent Suc-LLVY as a substrate was significantly reduced in 20-day old *Httex1Q120* IFMs, which can be rescued by CRTC^OE^ (Fig. 6c). Miltefosine (MFS), an alkylphosphocholine inhibiting PI3K/Akt [69], is a positive hit in our compound screening. AKT inhibition was shown to increase CRTC activity via Salt induced kinase (SIK) [11, 70]. Flies fed with MFS (5 mg/ml) in conventional food for 24 h can increase CRE-LUC activity in IFMs (Supplementary Fig. 7c). Moreover, MFS administration (5 mg/ml, 5d) significantly rescues proteasome activity in 20-day old *Httex1Q120* IFMs, similar results were obtained with another positive hit in the screening, GDC0941(Pictilisib), also a potent PI3K inhibitor [71](Fig. 6d).

Chaperone mediated protein refolding capacity can be examined by luciferase renaturation assay [72]. After heat inactivation at 42 °C, luciferase undergoes progressively denaturation when incubated with thorax lysates, and nearly 50% luciferase activities were retained at 5 min (Supplementary Fig. 7d). Intriguingly, *Httex1Q120* IFMs have drastically weaker Luc activity after 5 min denaturation, which can be rescued by CRTC overexpression (Fig. 6e**)**.

Moreover, climbing ability and survival rate of *IFM-Gal4/UAS-Httex1Q120* flies showed significant reductions that was rescued by CRTC^OE^ (Fig. 6f–h). However, the climbing ability rescue effect was abolished on CREB deletion mutant background(CREB^Δ36^) (Fig. 6g**)**.

Together, these results indicated that genetic or pharmacological increase of CREB activity promotes proteostasis recovery and reduce HD pathogenesis.

### Increasing CREB activity rescues age related protein aggregates in muscles

**Fig. 7:**
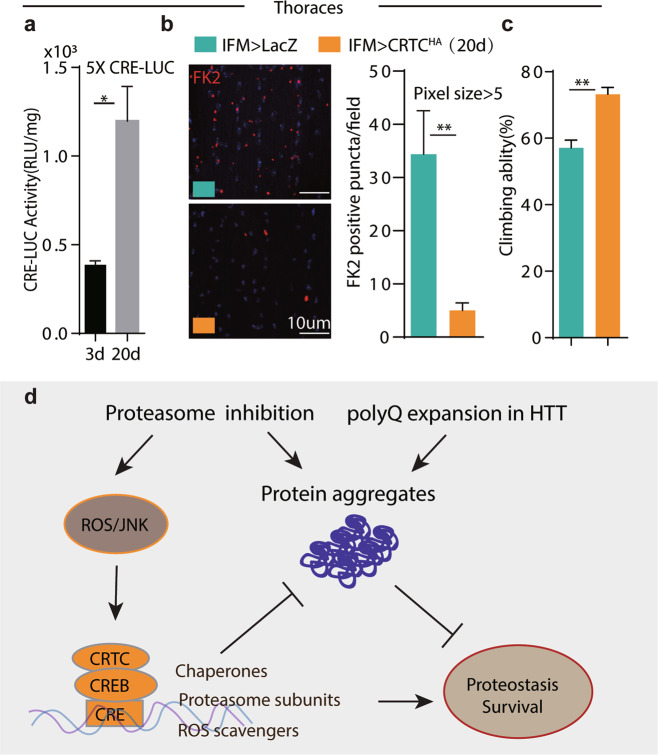
CRTC overexpression suppresses protein aggregates accumulated in aged muscles. **a** CRE-Luc activity in thoraces increased during aging. LUC activity at 3d and 20d-old thoraces were compared. *n* = 6 for each condition. Student’s *t*-Test for statistical analysis. *P < 0.05. Genotype: 5xCRE-LUC. **b**, **c** Age associated FK2 positive aggregates were ameliorated by CRTC overexpression. FK2 positive aggregates in 3d and 20d-old muscles were quantified. Representative confocal images were shown on the left (**b**). FK2 positive aggregates with pixel size bigger than 5 were quantified on the right (**c**). Student’s t-test for statistical analysis. **p < 0.01. Genotypes: *IFM-Gal4; UAS-RFP or IFM-Gal4; UAS-CRTC-HA*. **d** CRTC/CREB functions downstream of ROS/JNK pathway as a sensor to suppress proteotoxic stresses. Genetical or pharmacological inhibition of proteasome causes CREB activation through ROS/JNK cascade in flies. Together with CRTC, CREB promotes protein folding and proteasome activity by increase transcription of redox regulators, such as molecular chaperones, proteosome subunits, and ROS scavengers. Hence, increasing CRTC/CREB activity reduces protein aggregates and rescues pathogenesis of protein conformational disorders, such as Huntington’s disease (HD). CRTC cAMP-regulated transcriptional co-activators, CREB cAMP Responsible Element Binding Protein, CRE cAMP responsive element.

Previous studies showed that ubiquitin positive protein aggregates accumulated in IFMs during aging [66]. Transcript levels of chaperones and proteasome subunits are robustly increased in control IFMs by CRTC overexpression (Supplementary Fig. 7e). We then tested the role of CRTC/CREB during muscle aging. Interestingly, CRE-Luc activity in IFMs increased nearly two folds in 20-day-old flies compared with 3-day-old ones (Fig. 7a). Further increasing CREB activity by CRTC^OE^ significantly reduces protein aggregates (revealed by FK2 antibody) accumulated in control IFMs as well as climbing ability during aging (20-day-old) (Fig. 7b, c).

## Discussion

Proteasome dysfunction is a hallmark of aging, and correlates with many neurodegenerative diseases. In this study, we found proteasome inhibitors promote CREB activity through ROS/JNK signaling cascade. Our further studies identified CRTC/CREB function as a novel branch of unfolded protein response to cope with proteotoxic or oxidative stresses (Fig. 7d).

In mammals, CREB activity is mainly determined by its phosphorylation status [9]. In addition to PKA, stress induced kinases, such as p38, can change the phosphorylation status of CREB [12]. However, recent studies showed that CREB in Drosophila (dCREB) was constitutively phosphorylated under basal conditions, the nuclear abundance of CREB was a rate-limiting step for its activation [14]. It’s possible that the stability or nuclear entry of CREB could be regulated by JNK via phosphorylation at multiple sites. How dCREB activity is regulated by JNK needs further studies in the future. In mammalian cell lines, stimuli such as DNA damage, mitogens, cytokines can phosphorylate and activate CREB through p38 [73, 74]. Here we found JNK is required for proteotoxic stresses induced CREB activation in 293 T cells. These results suggested that upstream stimuli act on specific MAPKs to activate and fine-tune CREB dependent transcription. Comparing the transcriptional profile of CREB activation in response to p38 or JNK would be intriguing to explore in the future.

Huntingtin aggregates impair proteasome activity, while studies have also showed that aggregates played a protective role in HD. Protein aggregates can act as an adaptive mechanism to store toxic fragments before proteasome degrades them [75, 76]. Previous literatures showed that HDAC inhibitors can rescue HD pathogenesis in Drosophila [36], and Htt protein can sequester CBP, the coactivator of CREB, in mammals [35]. However, what aspects of CREB mediated transcription are compromised by Htt remains unknown. Here, we found CRTC/CREB facilitates protein folding and accelerates proteasomal degradation of aggregates by transcriptionally increasing related genes. However, the pathogenesis of HD is very complicated, other targets regulated by CRTC/CREB, such as mitochondrial function and metabolism, might also contribute to the rescuing effect. As a proteotoxic sensor, boosting CREB activity would serve as a potential therapeutic strategy for protein aggregation related diseases, such as HD.

## Materials and methods

### Fly food and husbandry

*IFM-GAL4* was generously provided by M, Guo, CaspermitoGrxRoGFP from T. Dick, *TORC*^*25-3*^ from M. Montminy, *NP1-GAL4* from D. Ferrandon, *UAS-CRTC:HA* from Y. Hiran. *GSTD:GFP, ARE:RFP* and *TRE:RFP* are originally from D. Bohmann lab. *tubulin-GS* from S. Pletcher, *UAS-tdTomato-P2A–GCaMP5G* from R.W. Daniels and 5966^*GS*^ from H. Jasper lab. *W*^*1118*^, *Da-GAL4, Tub-Gal80*^*ts*^, *UAS-prosβ5*^*RNAi*^ (34810), *5xCRE-LUC* (79016), *UAS-CREB*^*DN*^ (7219), *UAS-Rpt6*^*RNAi*^ (34712), *UAS-Rpn3*^*RNAi*^ (34561), *mChFP-Rho1*(52281), *UAS-hep[act]* (9306), *UAS-CREB*^*RNAi*^ (63681), *CREB*^*Δ36*^ (79018) and *UAS-HTT.ex1.Q120*(68408) from Bloomington Drosophila Stock Center. *UAS-Ero1L*^*RNAi*^ (TH04728.N), *UAS-Perk*^*RNAi*^ (THU4905), and *UAS-Ire1*^*RNAi*^ (THU1832) are from Tsinghua Fly Center.

Flies were cultured and maintained at 25 ˚C, 60% humidity with a 12 h: 12 h light-dark cycle. Flies were cultured on yeast/molasses-based standard fly food (recipe: 10 L H2O, 138 g agar, 220 g molasses, 750 g malt extract, 180 dry yeast, 800 g corn flour, 100 g soy flour, 62.5 ml propionic acid, 20 g Methyl 4-Hydroxybenzoate, and 72 ml ethanol).

### Luciferase assays

CRE Luciferase activity was measured with the Steady-Glo Luciferase Assay Kit (Promega Cat# E2510) based on the manufacture instruction. In brief, whole flies or tissues were freshly homogenized in 100 μl Glo lysis buffer. After centrifuged at 12,000 × *g* for 10 min, 30 μL supernatant were aliquoted in triplicates in 96-well plates. Three independent samples of each condition were analyzed. After incubation for one minute in dark, luminescence value was measured by a microplate reader (Synergy HTX, BioTek, Winooski, Vermont, USA). Luminescence values were then normalized with protein concentrations, which were determined with BSA as a standard using a bicinchoninic acid (BCA) protein determination kit (YEASEN

Cat#20201ES76) according to the manufacturer’s instructions.

### Large scale compound screening

U-GLAD system was utilized for large-scale compound screening in flies [19]. In brief, a compound library containing 1508 FDA approved drugs and 345 natural products (DiscoveryProbe^™^ FDA-approved Drug Library, ApexBio Cat# L1001) were individually mixed in Gum Arabic and dissolved in chemically defined liquid food to form micelles (final concentration: 5 mg/ml). These micelles are then delivered to CRE-LUC flies in vials by a U-shape glass capillary. Chemically defined food recipe was based on previous study [37]. CRE-Luciferase activity was measured using the Steady-Glo Luciferase Assay Kit (Promega). Protein concentrations were determined with BSA as a standard using a bicinchoninic acid (BCA) protein determination kit (YEASEN Cat#20201ES76) according to the manufacturer’s instructions.

### Drosophila food intake measurement

Food intake was measured by the capillary feeder assay (CAFÉ) with modifications [77]. Around 15–20 sex-matched flies (3-4d old) were dry starved for 4 h before feeding with liquid food via the U-GLAD system [19]. The amount of liquid food consumed by flies was measured after 1 h, food was colored with blue food dye (Erioglaucine disodium salt, MACKLIN, Cat#3844-45-9) for visualization. The volume decrease at each time point was calculated.

### Immunostaining and microscopy

Immunostaining was performed based on previous publication [15]. In brief, tissues were first dissected in 1X PBS (for guts) or in 4%formaldehyde (for thoraces), then fixed for 45 min at room temperature in 4%formaldehyde. After wash for 1 h in washing buffer (PBS, 0.5% BSA, 0.1% Triton X-100), tissues were incubated with primary antibodies and secondary antibodies diluted in washing buffer. Samples are then mounted and imaged with Zeiss AxioImager M2 with the apotome system. Images were then processed with ZEN and Image J software. Antibodies used in the studies:

rabbit anti-pH3 (Cat#06-570, Sigma-Aldrich) 1:1000,

rabbit anti-CREB (Cat#9197, Cell Signaling Technology), 1:800,

mouse anti-FK2(Cat#ENZ-ABS840-0100, Enzo Life Sciences),1:300,

mouse anti- Prospero (Cat# MR1A, Developmental Studies Hybridoma Bank, DHSB),1:100

rabbit anti-phospho-eIF2α(#Y407807, Applied Biological Materials),1:400,

anti-GAPDH (YEAEN, Cat#30210ES60) 1:2000.

### Quantitative real-time PCR

RNA was isolated from brains, or guts of 15 flies using TriZol reagent (Life technologies). Around 20 ug RNA then were reversely transcribed using 5X All-In-One RT Mastermix With Accurt Kit (#G592, Applied Biological Materials) according to the manufacturer’s instructions. Real-time PCR was performed with a CFX96^TM^ Real-Time System (Bio-Rad Laboratories). Transcription values were normalized with *Rp49*. Primers included:

RP49(Forward:TCCTACCAGCTTCAAGATGAC,Reverse:CACGTTGTGCACCAGGAACT),

RPN3(Forward:CGGAGGATGCCGAGTTTATT,Reverse:GCTGTAGATGTCCGTACTTTCC),

RPT6(Forward:CTACCATAAAGGCGAGGGATTC,Reverse:CCTGCAGTCGTCTCAAATTCT),

PERK(Forward:CCTCTCCTTGACGACGTTATT, Reverse:CTCATGCTGACCTACGCTAAA),

HSP26(Forward: CGTGCTCACCGTCAGTATTC,Reverse: CCTCGCTTTCATTTGCCTTAAC),

HSP27 (Forward: CTGGAGGATGACTTCGGTTT, Reverse: CCTCTCGTACGGCGAATAAC),

Hsp70Bb (Forward: TGTGCTCCGCATCATCAAT, Reverse: CCGCCCAAGTCGAAGATAAG),

GstD1 (Forward: CCAGGTGTATTTGGTGGAGAA, Reverse: GAAGTACAGGCGCTGATTGA),

Jafrac1 (Forward: TTCTTCTACCCGCTGGACTT, Reverse: CGATCACCTCGCAATTGATCTT),

Trxr-1 (Forward: GGCCTGTCTGGATTTCGTTAAG, Reverse: TGCATCAGCTTCTTGGGAATG),

CrebB (Forward: GATACAGGCCAATCCCTCGG,Reverse: GTGTGGATGACCGTCGAGTT),

PUC (Forward: CCTAGCAATCCTTCGTCATCTT,Reverse: TCGCTATCCGACTTGGATTTAC),

upd3 (Forward: GCACCAAGACTCTGGACATT,Reverse: GAAGGTTCAACTGTTTGCTAGTG),

GstD8 (Forward: AGAAGAAGGCTGTGGTCAATC,Reverse: GGATCGGCGGGATGATTATT),

ERO1L(Forward:ACAACGAGACGGCTAACAAG, Reverse:CTAGAGTGCAGGCCAGAAATAA).

### ROS measurement

For DHE staining, guts were dissected in Schneider’s medium, incubated in 30 µM DHE (Cat# D11347, Invitrogen) for 5 min at room temperature in the dark. After washed twice in 1XPBS, the samples were mounted and imaged immediately. Images were captured immediately via Zeiss AxioImager M2 with the apotome system (543 nm excitation, 550–610 nm detection).

For roGFP-based biosensor, adult fly guts were dissected in the presence of 20 mM N-ethyl maleimide (NEM) (Cat# E387, Sigma-Aldrich) [34]. All samples were further incubated with NEM for 10 min at room temperature (RT). Remaining NEM was removed by rinsing once with 1x PBS. Afterward, samples were fixed with 4% PFA for 15 min at RT. Remaining PFA was removed by washing twice with PBS for 10 min. Samples were equilibrated in glycerol mounting medium overnight at 4 °C and mounted the next day. Samples were stored horizontally at 4 °C. Probe fluorescence was excited sequentially at 405 and 488 nm and detected at 500–530 nm.

### Climbing ability assay

Climbing assay is based on previous publications [78]. Briefly, grouped flies were tested in 20 cm climbing vials. The number of flies that could climb to the top of the vial after 10 s was counted. Each vial was tested 10 times with 1 min of rest between tests and these were averaged as technical replicates.

### Lifespan experiments

1 or 2-day-old males and females were kept together overnight to ensure the same mating status. They were then separated by sex and subjected to the desired experimental treatment. Around 50 flies in each bottle were cultured and flies were transferred to fresh food three times per week. Flies were scored daily as alive or dead till the last survivor was dead. Three independent experiments were performed for each genotype. Statistical analysis is performed using GraphPad Prism 8 and Log-rank tests were performed for survivorship.

### Survival assays after stress treatment

3-day-old flies with indicated genotypes were transferred to vials containing 5% sucrose supplemented with 5 mg/ml Paraquat (with 20 flies per vial) or MLN2238(5 mg/ml) in EtOH. The numbers of dead flies were counted daily. Three independent experiments with around 60 flies were performed for each genotype.

### Statistical analysis

Statistical analysis is performed using GraphPad Prism 8 and indicated in figure legends. Layout of all figures used Adobe Illustrator CC2019. Experimental flies and genetic controls were tested at the same condition, and data are collected from at least three independent experiments. Mean and S.E.M. were shown.

### 26 S Proteasome chymotrypsin-like activity assay

Fly thoraces lysates were homogenization in assay buffer (50 mM Tris-HCl, 5 mM MgCl_2_, 1 mM DTT) and incubated at room temperature for 30 min. Enzyme activity was initiated by supplementing with 100uM of a fluorogenic substrate specific for chymotrypsin-like activity (Suc-LLVY-AMC) (Enzo Life Sciences, Cat# P802) and 1 mM ATP in assay buffer to measure 26 S activity. Fluorescence of the liberated AMC substrate was measured using a microplate reader (Synergy HTX, BioTek, Winooski, Vermont, USA). The fluorescence emission at 460 nm was obtained after 1 hr at the excitation wavelength of 360 nm.

### Protein refolding capacity

Protein refolding activity was examined with luciferase inactivation assay with minor modifications [72]. In brief, 100 nM luciferase (Sigma-Aldrich Cat#61970-00-1) was heated at 42 °C alone or in the presence of the fly muscle lysates in luciferase refolding buffer (LRB: 25 mM HEPES-KOH at pH 7.4, 150 mM potassium acetate,10 mM magnesium acetate and 10 mM DTT). Luminescence was measured on a microplate reader (Synergy HTX, BioTek, Winooski, Vermont, USA) at different time points (5, 10, 15 min). Luminescence values were then normalized with protein concentrations using BSA as a standard by bicinchoninic acid (BCA) protein determination kit (YEASEN Cat#20201ES76) according to the manufacturer’s instructions.

### RNA-seq analysis

For RNAseq in fly guts, around 20 guts of each sample were dissected in RNase-free PBS and placed in Trizol. Extracted RNA and cDNA library was generated as described previously [79]. Sequencing was performed using an Illumina HiSeq2000 machine. Data were analyzed with OmicShare software. Metadata files were submitted to the Gene Expression Omnibus repository (GEO), with GEO accession number (GSE185159).

### Mammalian cell culture

HEK-293T cells were cultured in Dulbecco’s modified Eagle’s medium (DMEM, Gibco) supplemented with 10% fetal bovine serum, 10% Glutamine and 1% penicillin/streptomycin in an incubator at 5% CO_2_ and 37 °C.

For immunofluorescence study, cells were plated on 12 mm round coverslips in 24-well dish. After compound incubation (40 uM SP600125 or 5 nM MLN2238 for 24 h), cells were washed three times with ice-cold 1XPBS, then fixed for 15 min at room temperature with 4%formaldehyde, and then permeabilized Triton X-100 for 20 min. Following permeabilization, nonspecific binding in the cells was blocked for 1 h at room temperature. Then cells were incubated for 1 h with primary antibodies.

After three washes with 1XPBS, the cells were incubated for another 1 h with secondary antibodies.

Primary antibodies included:

rabbit anti-CREB (Cat#9197, Cell Signaling Technology), 1:800

rabbit anti- Phospho-CREB (Cat#9198, Cell Signaling Technology), 1:800

mouse anti-JNK 1:500 (Y061991, ABMgood)

Secondary antibodies were anti-mouse CY3 (1:500) and anti-rabbit CY5 (1:500).

For Western Blot, cells were washed by ice-cold 1XPBS, then lysed with RIPA lysis buffer supplemented with protease inhibitor and phosphatase inhibitor cocktail on ice for 30 min. Cell lysates were then cleared by centrifugation at 15,000 rpm for 10 min at 4 °C. Protein were analyzed on 10%SDS-PAGE with anti-CREB (Cat#9197, Cell Signaling Technology), rabbit anti-Phospho-CREB (Cat#9198, Cell Signaling Technology). Anti-α-tubulin (Beyotime, Cat#AT819) were used as loading controls.

### Triton-Insoluble protein extracts and Western blot

Western blots of insoluble fractions were obtained substantially as described before [5]. Briefly dissected thoraces were homogenized in ice-cold 1XPBS with 1% Triton X-100 and protease inhibitors. The mixture was centrifuged at 14,000 rpm for 10 min at 4 °C, and the pellet and supernatant were collected. The remaining pellet was washed in Triton X-100 buffer and centrifuged twice at 14,000 rpm for 5 min at 4 °C. The pellet was then resuspended at room temperature in 100 ul RIPA buffer, centrifuged at 14,000 rpm at 4 °C for 10 min, and the supernatant collected (Triton X-100 insoluble fraction). Insoluble fractions were analyzed on 10%SDS-PAGE with anti-ubiquitin (Cell Signaling Technologies P4D1, Cat #3936). anti-GAPDH (YEAEN, Cat#30210ES60) were used as loading controls.

### Pharmacological treatment in flies

To turn on GeneSwitch system in 5966Gal4^GS^ and *tubulin*Gal4^GS^ containing flies, RU486 (mifepristone) (MACKIN, Cat# M830038) was dissolved in ethanol. 200 µl of a 5 mg/ml solution of RU486 in 80% ethanol was deposited on surface of the conventional food. Equal amount of 80% ethanol only solution was used as mock control. The food was then dried for at least 16 h to ensure complete evaporation. Flies kept at 25 °C were fed on RU486 or mock food for 24 h and dissected at 4-6days after treatment.

Information of other compounds:

Lipoic acid, 2 mM (in EtOH) Sigma-Aldrich Cat# T5625; CAS: 1077-28-7.

PBA (Sodium phenylbutyrate), 20 mM (in H_2_O) Sigma-Aldrich Cat# SML0309; CAS: 1716-12-7.

NAD (Nicotinamide adenine dinucleotide), 10 mM (in DMSO) Sigma-Aldrich Cat# N0632; CAS: 20111-18-6.

NAC(N-Acetyl-L-cysteine), 1 mg/ml (in H_2_O) Sigma-Aldrich Cat# A7250; CAS: 616-91-1.

MLN2238,5 mg/ml (in EtOH), APExBIO Catalog No. A4008.

Paraquat 5 mg/ml (in H_2_O) (1,1′-dimethyl-4,4′-bipyridinium dichloride) Sigma-Aldrich CAS:75365-73-0.

## Supplementary information

aj-checklist

Original data for anti-CREB staining in 293T cells

Supplemental Figure legend

Supplemental Figure 1

Figure S2

Supplemental figure 3

Figure S4

Supplemental figure 5

Supplemenal figure 6

Figure S7

Supplemental table 1

supplemental table 2

## Data Availability

All sequencing datasets generated in this study are freely available through the Gene Expression Omnibus (GEO), with accession number GSE185159. All data generated or analyzed during this study are available from the corresponding author upon request.
