# Supplementary figures and images for: The CRTC-CREB axis functions as a transcriptional sensor to protect against proteotoxic stress in Drosophila
Source: Cell Death Dis. 2022 Aug 6;13(8):688. doi: 10.1038/s41419-022-05122-y (PMC9357022; doi:10.1038/s41419-022-05122-y)

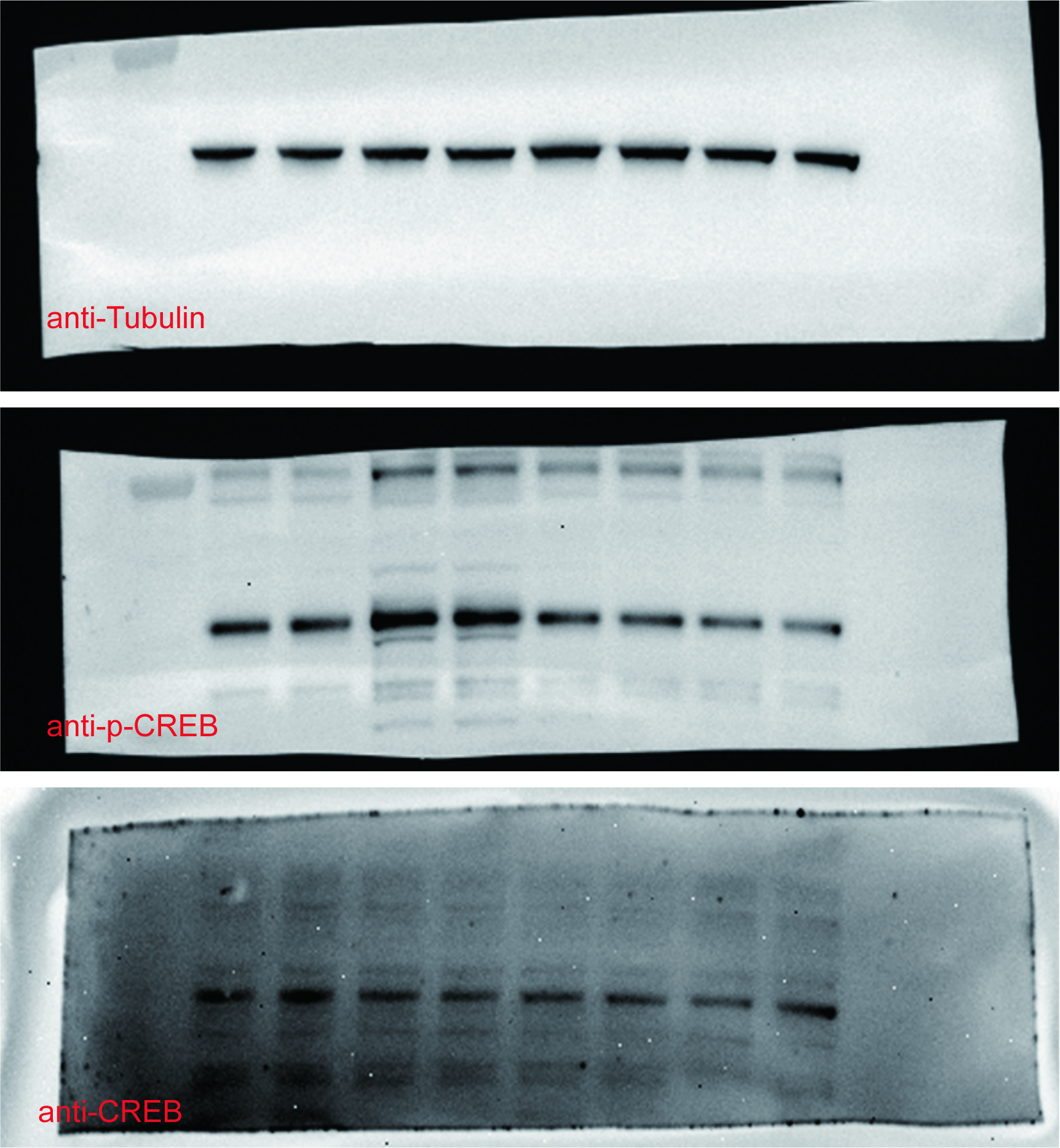

Supplement: Supplementary file 2 — Original data for anti-CREB staining in 293T cells [file 41419_2022_5122_MOESM2_ESM.tif]

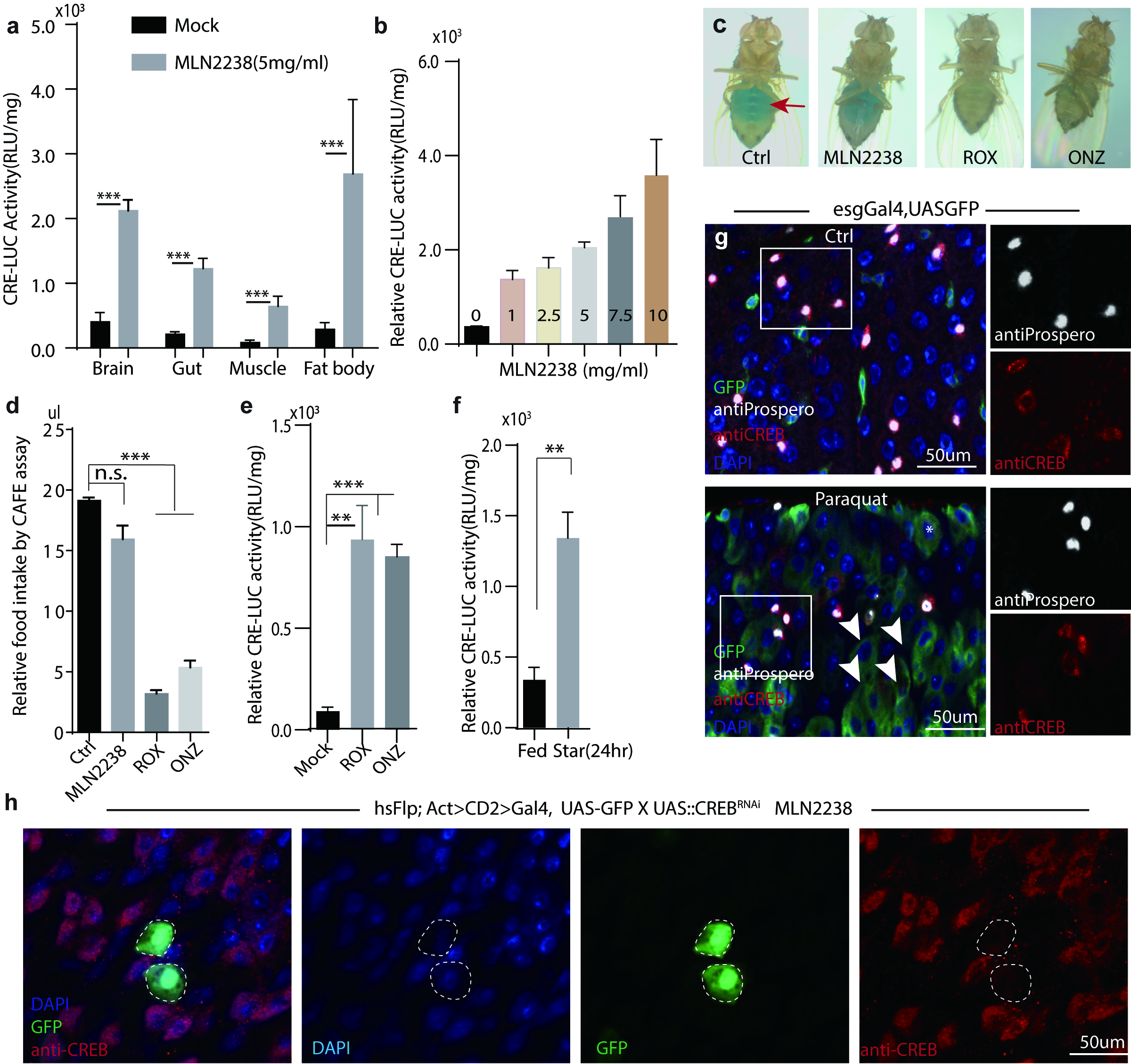

Supplement: Supplementary file 4 — Supplemental Figure 1 [file 41419_2022_5122_MOESM4_ESM.tif]

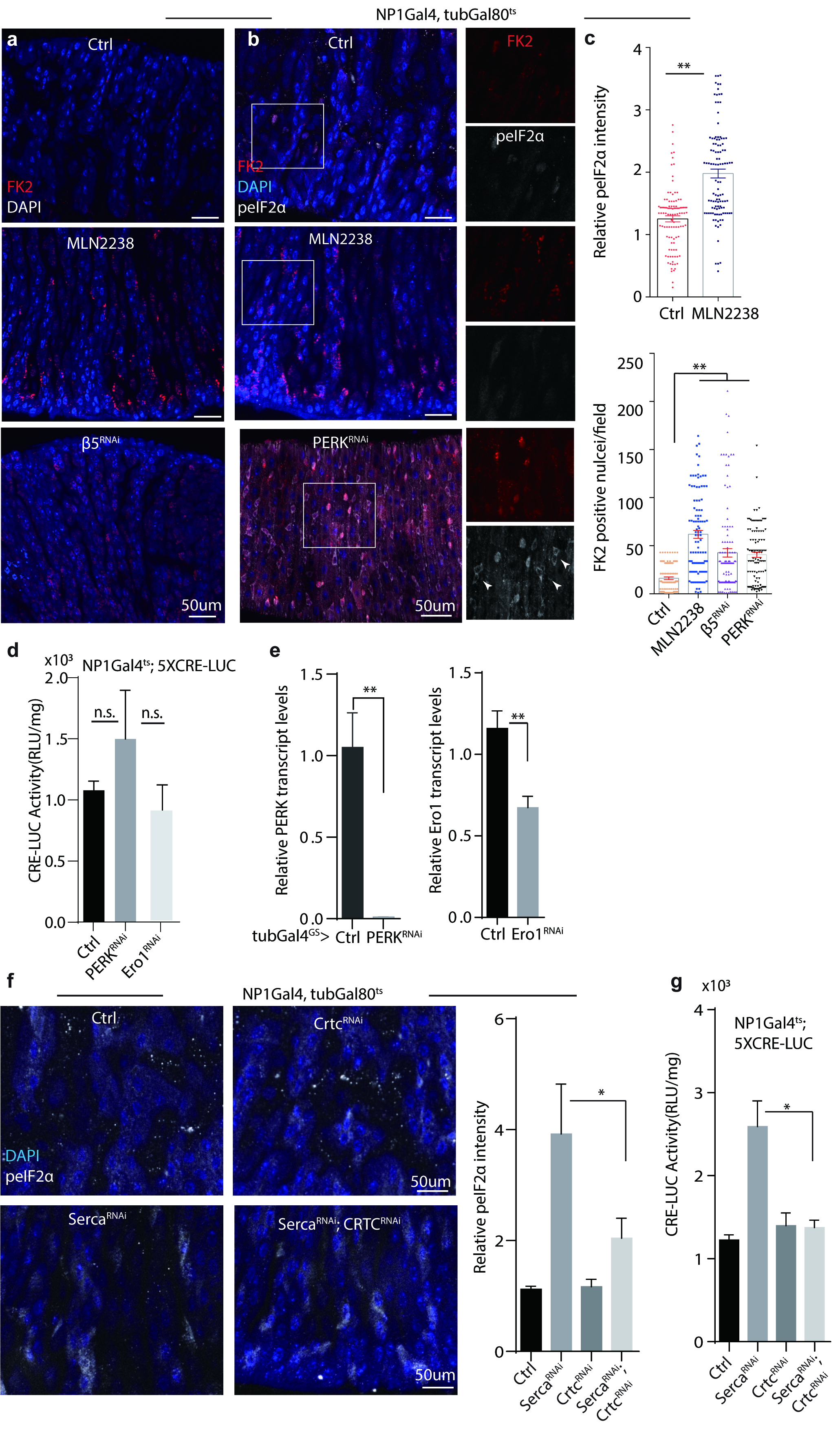

Supplement: Supplementary file 5 — Figure S2 [file 41419_2022_5122_MOESM5_ESM.tif]

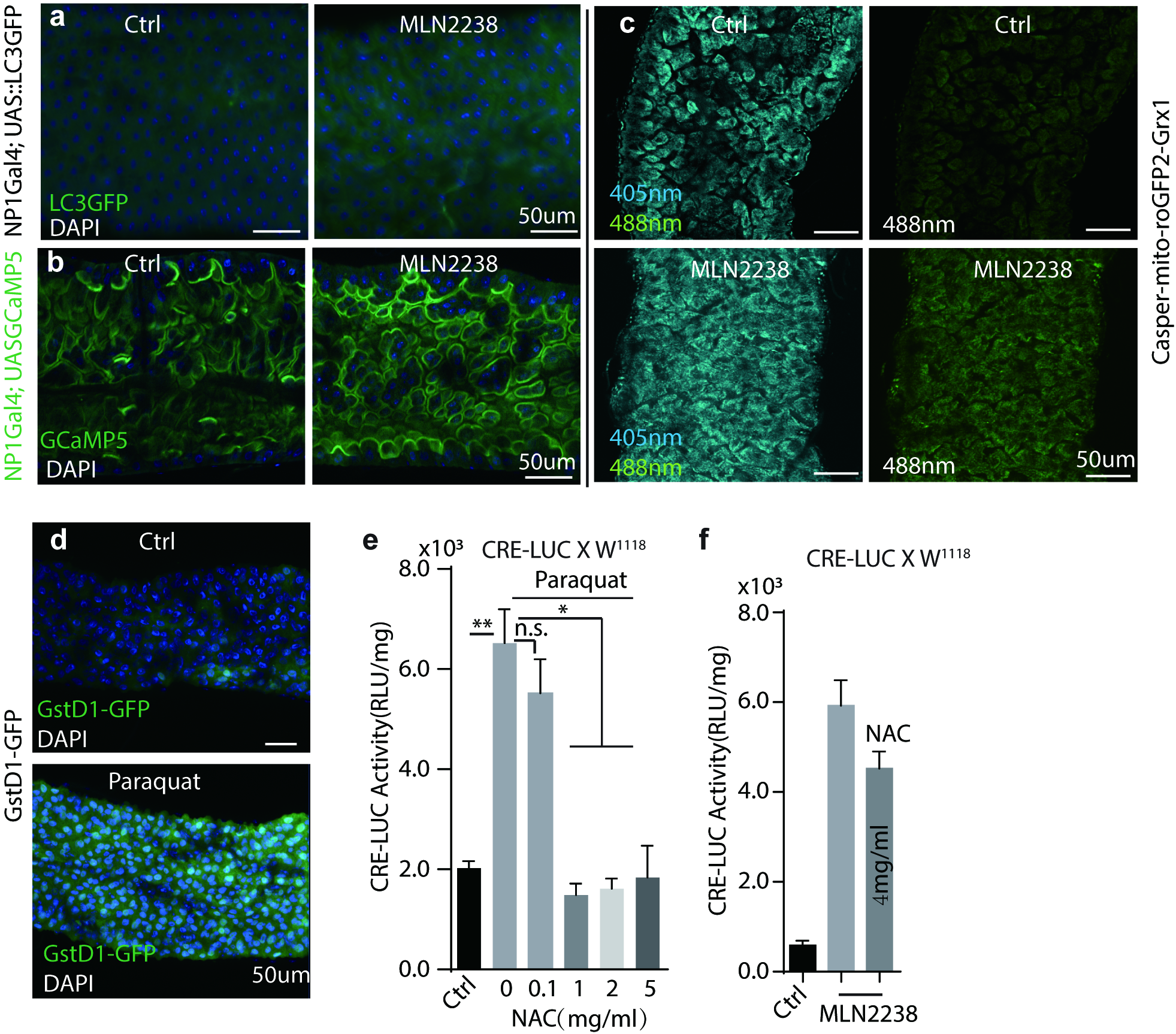

Supplement: Supplementary file 6 — Supplemental figure 3 [file 41419_2022_5122_MOESM6_ESM.tif]

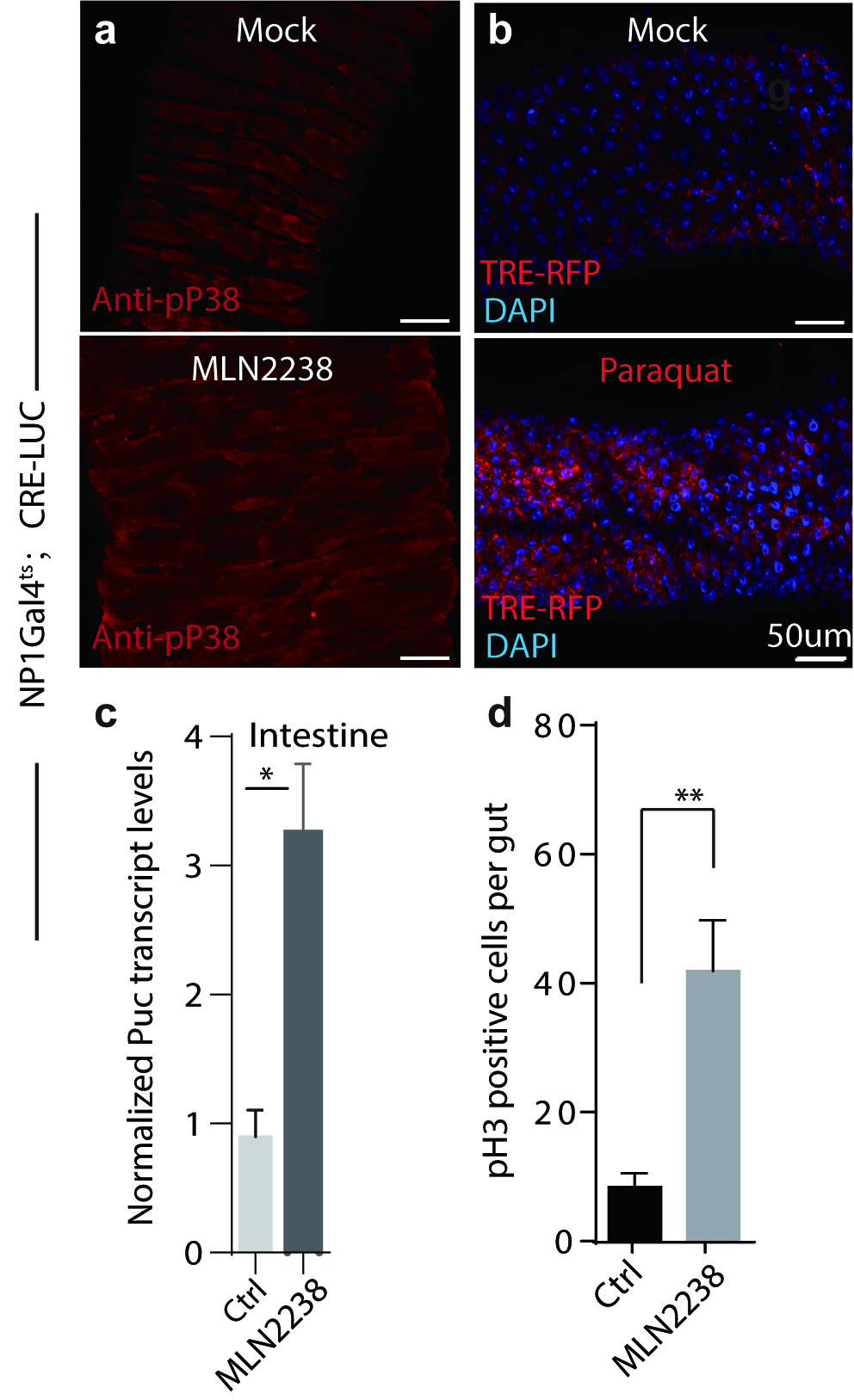

Supplement: Supplementary file 7 — Figure S4 [file 41419_2022_5122_MOESM7_ESM.tif]

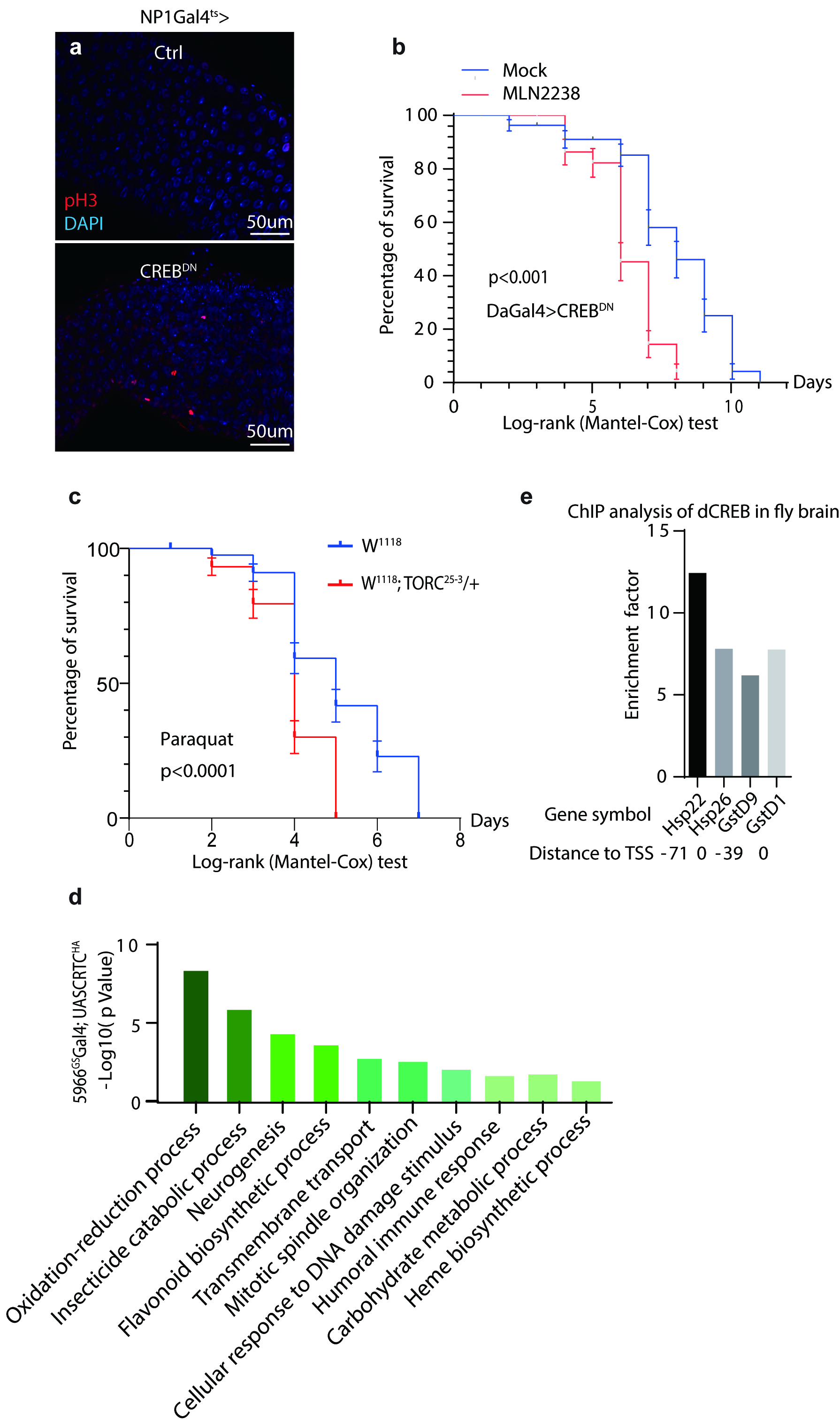

Supplement: Supplementary file 8 — Supplemental figure 5 [file 41419_2022_5122_MOESM8_ESM.tif]

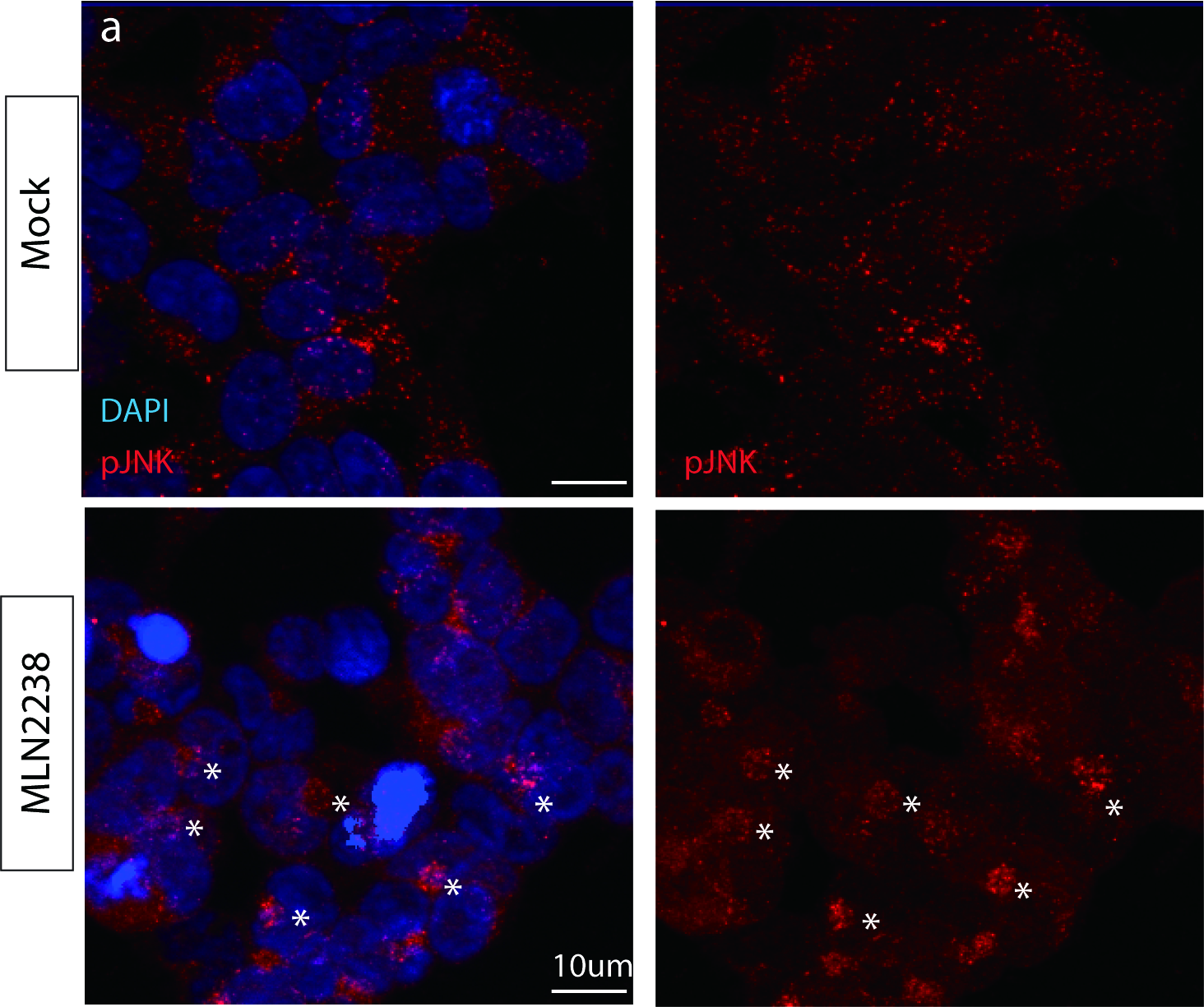

Supplement: Supplementary file 9 — Supplemenal figure 6 [file 41419_2022_5122_MOESM9_ESM.tif]

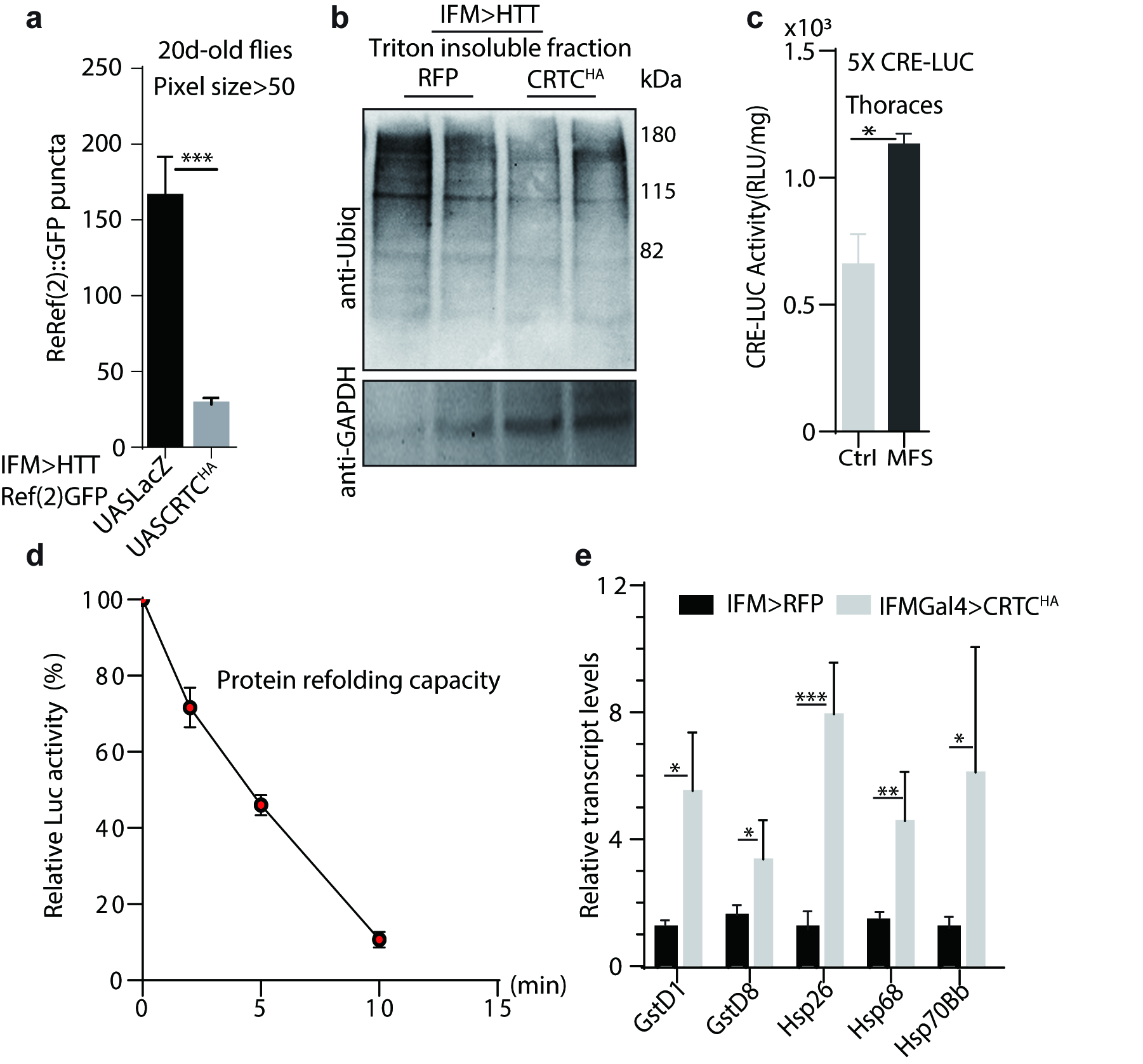

Supplement: Supplementary file 10 — Figure S7 [file 41419_2022_5122_MOESM10_ESM.tif]
