# Supplementary material for: The CRTC-CREB axis functions as a transcriptional sensor to protect against proteotoxic stress in Drosophila
Source: Cell Death Dis. 2022 Aug 6;13(8):688. doi: 10.1038/s41419-022-05122-y (PMC9357022; doi:10.1038/s41419-022-05122-y)
Supplement: Supplementary file 3 — Supplemental Figure legend [file 41419_2022_5122_MOESM3_ESM.docx]

**Supplemental figure S1: Proteasome inhibitors promote CREB activity in adult flies.**

**a**, MLN2238 treatment (5mg/ml, 24hr) increase CER-LUC activity in different tissues. Student’s t-test performed for statistics. *** *P*<0.001.

**b**, Dosage dependent effects of MLN2238 on CREB activity (CRE-LUC) in adult flies were shown. Concentrations were shown inside the columns. n=5 for each condition.

**c**, Representative images of flies ingested with compounds mixed in blue-dyed food.

**d,** Food intake rate of indicated compounds(5mg/ml) was monitored by CAFÉ assay. n=20 for each condition. Student’s t-test for statistical analysis. n.s*.*: no significance. ***: *P*<0.001. ROX: Roxithromycin, ONZ: Ornidazole.

**e-f**, ddH2O starvation mimics the effect of antibiotics on CRE-LUC activity in flies. n=5 for each condition. Student’s t-test for statistical analysis. **: *P*<0.01. ***: *P*<0.001.

**g**, CREB expression in adult fly guts was examined. GFP labels progenitor cells in green, Prospero labels enteroendocrine cells in gray. CREB signals(red) detected with antibody against CREB staining. Separate channels shown on the right. Arrowheads denoted the typical mis-differentiated cells (GFP positive while differentiating). n=6 for each condition. Scale bars: 50um. Genotypes for **a-f**, 5xCRE-LUC, for **g**, *esgGal4, UASGFP*.

S.E.M for error bars.

**h,** CREB protein level was detected by immunostaining in FLIP-out clones of *CREB^RNAi^* after MLN2238 treatment (5mg/ml, 24hrs). CREB was specifically knocked down in GFP positive cells, which were denoted by dashed circles. Separated channels were shown on right.

**Supplemental figure S2: Proteasome inhibition regulates CREB activity through ROS.**

**a**, Ubiquitinated proteins in fly guts were examined by FK2 staining(red). Nuclei were counter-stained by DAPI in blue.

**b**, Flies with indicated conditions were co-stained with anti-ubiquitin FK2 antibody and an antibody against p-eIF2α. Arrowheads point to p-eIF2α positive progenitor cells induced by *PERK^RNAi^*. Scale bars: 50μm. Genotypes for **a-b**: *NP1Gal4, tubGal80^ts^* or *NP1Gal4, tubGal80^ts^; UASProsβ5^RNAi^*, or *NP1Gal4, tubGal80^ts^; UAS-PERK^RNAi^*. Separate channels of boxed areas shown on the right.

**c**: Upper panel, relative peIF2α level in enterocytes were quantified after MLN2238 treatment. DAPI signals were used as internal control. At least 100 enterocytes from 5 guts of each condition were quantified. Mann Whitney U test for statistical analysis. **:p<0.01. Genotype: *NP1Gal4, tubGal80^ts^.*

Lower panel: number of FK2 positive puncta was quantified after MLN2238 treatment

or when β5 subunit or *PERK* was knocked down specifically in enterocytes. Kruskal-Wallis test with Dunn’s multiple comparison tests for lower panel. **:p<0.01. Genotypes: *NP1Gal4, tubGal80^ts^,* or *NP1-Gal^ts^; UAS-*β5 *^RNAi^, or NP1-Gal^ts^; UAS-PERK^RNAi^*.

**d**, CRE-LUC activity was measured when ER^UPR^ components were silenced in guts. Genotypes: *NP1-Gal^ts^, CRE-LUC or NP1-Gal^ts^, CRE-LUC; UAS-PERK^RNAi^ or NP1-Gal^ts^, CRE-LUC; UAS-Ero1L^RNAi^*. n=10 for each condition. Student’s t-test for statistical analysis. *n.s*. no significance.

**e**, Knock-down efficiency of *PERK^RNAi^* and *Ero1^RNAi^* was verified by RT-qPCR. Relative transcription was normalized with *Rp49*. Genotypes: *tubGal4^GS^; UASPERK^RNAi^ or tubGal4^GS^; UASEro1^RNAi^*. n=6 for each condition. Samples were run in triplicates. **: *P*<0.01, Student’s t-test for statistical analysis. S.E.M for error bars.

**f,** *SERCA* knocking down in enterocytes causes ER stresses. ER stresses status indicated by relative peIF2α level were analyzed. Representative images were shown. DAPI(blue), p-eIF2α(grey). Genotype: *NP1-Gal^ts^* or *NP1-Gal^ts^; UAS-SERCA^RNAi^* or *NP1-Gal^ts^; UAS-CRTC^RNAi^* or *NP1-Gal^ts^; UAS-SERCA^RNAi^, UAS-CRTC^RNAi^.* 10 guts of each condition were imaged. Quantification was shown on right. At least 100 enterocytes from 5 guts of each genotype were quantified. t-Test for statistical analysis. ***:*p*<0.001.

g, CRE-LUC activity in fly guts of indicated genotypes were quantified. T-Test were performed for statistically analysis. Biological triplicates were used. S.E.M was shown

*: p<0.05.

**Supplemental figure S3: Proteasome inhibition regulates CREB activity through ROS.**

**a**, Autophagic activity in guts (LC3GFP, green) after MLN2238 treatment was examined. Representative images were shown. Genotype: *NP1Gal4, UAS::LC3GFP*.

**b**, Cytosolic Ca^2+^ level in guts (GCaMP5, green) after MLN2238 treatment was analyzed. Representative images were shown. Genotype: *NP1Gal4, UASGCaMP5*.

c, Representative images of ROS levels after MLN 2238 treatment were shown by a ratio-metric sensor *Casper-mito-roGFP2-Grx1* with two different excitation wave-length (405 nm and 485 nm).

**d**, Oxidative stress reporter GstD1-GFP (green) after MLN2238 treatment were examined. In **a-c**, DAPI stains nuclei in blue. n=6-7 for each condition. Scale bars for **a-d**: 50μm.

**e**, The effect of NAC on CRE-Luc activity after paraquat treatment was analyzed. NAC were fed at indicated concentrations. Student’s t-test performed for statistical analysis. *n.s*. no significance, *: *P*<0.05, **: *P*<0.01. S.E.M for error bars.

f, NAC administration partially suppresses MLN2238 induced CRE-LUC activity in guts. Animals were fed with NAC(4mg/ml) for 4 days and then feeding together with MLN2238 for another 4 days. 15 guts were then dissected and measure using Steady-Glo Luciferase Assay Kit (Promega Cat# E2510). Three biological independent experiments were performed.

**Supplemental figure S4, related to Figure 3: JNK is downstream of proteasome inhibition to regulate CREB activity**

**a**, p38 activity in fly intestine was examined by immunostaining against phospho-p38 with or without MLN2238 treatment. Representative images were shown.

**b**, JNK activity (indicated by TRE-RFP) after paraquat treatment was shown. DAPI stains nuclei in blue. Scale bars: 50μm. At least 7 animals of each condition were analyzed.

**c,** Relative transcription of *puckered* by RT-qPCR. Experiments were performed in triplicates. Student’s t-test performed for statistical analysis. *n.s*. no significance, *: *P*<0.05.

d, Mitotic cells in gut were quantified by anti-pH3 staining. At least 10 animals of each genotype were analyzed and quantified. *S.E.M* was shown, T-test for statistically analysis. **: p<0.01. Genotypes: *NP1-Gal^ts^, CRE-LUC*.

For MLN 2238 treatment, files were fed with MLN2238 at 5mg/ml for 24hrs before dissection.

**Supplemental figure S5, related to Figure 4: CRTC/CREB is essential to maintain redox and proteostatic homeostasis.**

**a**, Representative images of pH3 staining in fly intestine. Genotypes: *NP1Gal^ts^; UASCREB^DN^.* Mitotic ISCs were stained by pH3 in red, DAPI stains nuclei in blue. Scale bars: 50μm.

**b-c**, Survival rate after MLN2238 (**b**) or paraquat (**c**) treatment was analyzed. *p*<0.0001, Log-rank (Mantel-Cox) test was performed for statistics. At least 50 animals for each condition were analyzed. Genotype for **b**, *DaGal4; UASCREB^DN^*, for **c**, *TORC^25-3^/+.*

**d**, KEGG analysis of transcriptional profiling results after CRTC overexpression in fly intestine. Top 10 gene functional categories upregulated by CRTC overexpression were shown. Genotype: 5966Gal4^GS^; UASCRTC^HA^.

e, Molecular chaperones and redox regulators are enriched in ChIPseq dataset for CREB in fly brain (GSE81456). Enrichment factor for typical genes are shown. Distance to the transcriptional start site(TSS) was shown on bottom.

**Supplemental figure S6 MLN2238 promotes JNK phosphorylation in 293T cells.**

a, Representative images of immunostaining against phospho-JNK( Thr^183^ and Tyr^185^) after MLN2238（5nM, 24 hrs）treatment in 293T cells. Separate channels shown on the right. Scale bar: 10um. Stars denote pJNK positive foci in cytosol.

**Supplemental figure S7: Increasing CREB activity rescues pathogenesis in a fly model of Huntington disease.**

**a**, Quantification of protein aggregates in indirect flight muscles of 20d-old flies. Genotypes: *IFMGal4,* *UAS-HTT.ex1.Q120; UASRef(2)GFP, UASLacZ* or *IFMGal4,* *UAS-HTT.ex1.Q120; UASRef(2)::GFP,UASCRTC^HA^.* Scale bars: 20um.

**b,** Insoluble protein aggregated in thoraces lysates of indicated genotypes were analyzed by western blot using an antibody against Ubiquitin. Antibody against GAPDH used as a loading control.

**c**, RE-LUC activity in thoraces was quantified after MFS (Miltefosine) treatment. MFS was fed at 5mg/ml for 24 hrs. Student’s t-test performed for statistical analysis. *: p<0.05, Genotype: *IFMGal4; 5xCRE-LUC.*

**d,** Luciferase recovery curve after denaturation at 42^o^C. Percentage of Luciferase activity after denaturation for certain timepoint was shown.

**e,** Relative transcription of genes in IFMs was quantified by RT-qPCR and normalized with *Rp49*. Genotypes: *IFMGal4; UASCD8RFP* or *IFMGal4; UASCRTC^HA^*. Student t-Test for statistical analysis. **: p<0.01. *: p<0.05. Samples were run in triplicates. S.E.M for error bars.
